# A new winter wheat genetic resource harbors untapped diversity from synthetic hexaploid wheat

**DOI:** 10.1007/s00122-024-04577-1

**Published:** 2024-03-07

**Authors:** Tally I. C. Wright, Richard Horsnell, Bethany Love, Amanda J. Burridge, Keith A. Gardner, Robert Jackson, Fiona J. Leigh, Aleksander Ligeza, Sigrid Heuer, Alison R. Bentley, Philip Howell

**Affiliations:** 1 The John Bingham Laboratory, NIAB, 93 Lawrence Weaver Road, Cambridge, CB3 0LE UK; 2 Life Sciences, University of Bristol, Bristol, BS8 1TQ UK; 3 Present Address: International Maize and Wheat Improvement Center (CIMMYT), El Batan, Mexico; 4 Present Address: Processors and Growers Research Organization (PGRO), The Research Station, Thornhaugh, Peterborough, PE8 6HJ UK; 5 Present Address: Research School of Biology, Australian National University, Canberra, ACT 2600 Australia

## Abstract

**Key message:**

The NIAB_WW_SHW_NAM population, a large nested association mapping panel, is a useful resource for mapping QTL from synthetic hexaploid wheat that can improve modern elite wheat cultivars.

**Abstract:**

The allelic richness harbored in progenitors of hexaploid bread wheat (*Triticum aestivum* L.) is a useful resource for addressing the genetic diversity bottleneck in modern cultivars. Synthetic hexaploid wheat (SHW) is created through resynthesis of the hybridisation events between the tetraploid (*Triticum turgidum* subsp. *durum* Desf.) and diploid (*Aegilops tauschii* Coss.) bread wheat progenitors. We developed a large and diverse winter wheat nested association mapping (NAM) population (termed the NIAB_WW_SHW_NAM) consisting of 3241 genotypes derived from 54 nested back-cross 1 (BC_1_) populations, each formed via back-crossing a different primary SHW into the UK winter wheat cultivar ‘Robigus’. The primary SHW lines were created using 15 *T. durum* donors and 47 *Ae. tauschii* accessions that spanned the lineages and geographical range of the species. Primary SHW parents were typically earlier flowering, taller and showed better resistance to yellow rust infection (*Yr*) than ‘Robigus’. The NIAB_WW_SHW_NAM population was genotyped using a single nucleotide polymorphism (SNP) array and 27 quantitative trait loci (QTLs) were detected for flowering time, plant height and *Yr* resistance. Across multiple field trials, a QTL for *Yr* resistance was found on chromosome 4D that corresponded to the *Yr28* resistance gene previously reported in other SHW lines. These results demonstrate the value of the NIAB_WW_SHW_NAM population for genetic mapping and provide the first evidence of *Yr28* working in current UK environments and genetic backgrounds. These examples, coupled with the evidence of commercial wheat breeders selecting promising genotypes, highlight the potential value of the NIAB_WW_SHW_NAM to variety improvement.

**Supplementary Information:**

The online version contains supplementary material available at 10.1007/s00122-024-04577-1.

## Introduction

With the human population growing and the climate changing, there is an urgent need to future-proof crops to maintain high yields under increasingly unpredictable growing conditions. Furthermore, these problems must be addressed while improving the sustainability of farming practices. A prerequisite for using plant breeding to address these challenges is genetic diversity (Swarup et al. 2021). In comparison to its progenitor species, genetic diversity has been narrowed in the modern hexaploid bread wheat (*Triticum aestivum* L.) genepool through first domestication and then selective breeding (Haudry et al. 2007). Bread wheat progenitor species harbor additional genetic diversity that could provide a solution to improving key traits such as pest and disease resistance, tolerance to abiotic stress (e.g., drought or heat) and resource-use efficiency in modern wheat (as reviewed by Leigh et al. 2022). A good proportion of this diversity can be recaptured from extended sources by targeted resynthesis of the historic inter-species hybridisations that led to the evolution of bread wheat.

Between 8500 and 9000 years ago, a primitive cultivated tetraploid wheat (genome AABB) hybridized with the diploid wild goatgrass *Aegilops tauschii* Coss. (DD) to form hexaploid bread wheat (AABBDD), whose cultivation spread globally to become a staple human food source (Levy and Feldman 2022). Due to the rarity of the natural hybridisation events that led to bread wheat speciation (Giles and Brown 2006), only a small portion of *Ae. tauschii* standing genetic diversity was captured in the bread wheat D-genome (Wang et al. 2013). This evolutionary genetic bottleneck highlights the potential value of accessing the untapped genetic reserves of the D sub-genome progenitor for modern wheat improvement (Singh et al. 2019). One route to exploiting this diversity is through the creation of Synthetic Hexaploid Wheat (SHW), which acts as a bridge for transferring beneficial genetic variation from *Ae. tauschii* to modern wheat (Li et al. 2018; Gaurav et al. 2022).

The formation of SHW typically involves fertilizing a tetraploid wheat floret with pollen from diploid *Ae. tauschii* and using embryo rescue followed by chromosome doubling to produce a viable hexaploid wheat, called the primary SHW. This approach to wheat resynthesis was first practiced in the 1940s (Britten and Thompson 1941; McFadden and Sears 1946). Since the 1980s, the International Maize and Wheat Improvement Center (CIMMYT) has created large numbers of spring-type SHW lines using extensive collections of *Ae. tauschii* (Dreisigacker et al. 2008). Subsequently, many SHW-derived varieties (synthetic derivatives), developed through crosses between primary SHW and elite lines, have been released across the world, particularly across Asia (Li et al. 2018). The increased allelic richness and improvements in a range of target traits have driven the popularity of synthetic derivatives in breeding and variety release. These characteristics include disease and pest resistance (Morgounov et al. 2018; Shamanin et al. 2019), drought tolerance (Mokhtari et al. 2022), heat stress (Cossani and Reynolds 2015) and yield improvements (Jafarzadeh et al. 2016). However, their impact has been lower in areas that predominantly grow winter wheat under more intensive production systems, such as the United Kingdom (UK) and Northern Europe. Genetic mapping populations can be developed to identify quantitative trait loci (QTLs) in progenitor wheat backgrounds linked to novel diversity (e.g., Wright et al. 2020). Multi-parent crossing schemes, such as nested association mapping (NAM) panels, are a useful means of increasing genetic mapping resolution within a mapping population while also capturing increased genetic diversity (Scott et al. 2020). Originally developed in maize (Yu et al. 2008), NAM panels contain a series of linked nested bi-parental crosses (termed hereafter ‘nested populations’), in which different donors (e.g., varieties, breeding lines, or landraces) are each crossed to a single common parent to form a large genetic mapping resource. NAM populations have been developed for several crop species, including tetraploid (Kidane et al. 2019) and hexaploid (Bajgain et al. 2016; Jordan et al. 2018; Wingen et al. 2017) wheat. Notably, Gorafi et al. (2018) developed a NAM population of 4300 genotypes (400 of which were genotyped), derived from back-crossing 43 SHW lines to the Japanese bread wheat cultivar ‘Norin 61’. Identification of novel QTLs within NAM resources can be used for Marker-Assisted Selection programs in breeding or for further investigations such as gene discovery.

While representing rich sources of genetic variation, primary SHW lines themselves typically show many undesirable wild characteristics and are generally unadapted to targeted agricultural environments (Dreisigacker et al. 2008). This can be overcome by back-crossing into adapted and elite wheat backgrounds. Furthermore, backcrossing can be targeted such that introgressions from SHW are transferred into specific genomic regions of elite wheat cultivars (Horsnell et al. 2023). Here, we report the creation of a large winter wheat NAM resource, called the NIAB_WW_SHW_NAM population. The resource is composed of 54 primary SHW lines backcrossed into a single UK winter wheat genetic background. Through genotyping and field screening, this population was used to identify QTLs for important agronomic traits. Representatives from UK wheat breeding companies have evaluated the population and made selections of genotypes for their own breeding programs. The resource and associated data are openly available to support further interrogation of genetic variation from SHW and for use in trait discovery and pre-breeding.

## Materials and methods

### Material and generation of the primary SHW

From 2012 to 2017, NIAB created 37 primary Synthetic Hexaploid Wheat (SHW) lines, capturing 34 *Ae. tauschii* and 5 *Triticum turgidum* subsp. *durum* Desf. parental accessions. Additionally, 10 primary SHW lines were obtained from the Academy of Agricultural and Forestry Sciences (NARDI), Fundulea, Romania and seven primary SHW lines were obtained from the International Maize and Wheat Improvement Center (CIMMYT), Ankara, Turkey. These 17 genotypes were developed using 13 unique *Ae. tauschii* accessions and 10 unique *T. durum* accessions. In total, 54 primary SHW lines were available for use in NAM population formation, originating from 15 *T. durum* and 47 *Ae. tauschii* accessions. The sampling origins and sources of these materials are shown in Supplementary Table S1. The methodology for the creation of the NIAB primary SHW lines is described in Gaurav et al. (2022).

### NIAB_WW_SHW_NAM population formation

**Fig. 1:**
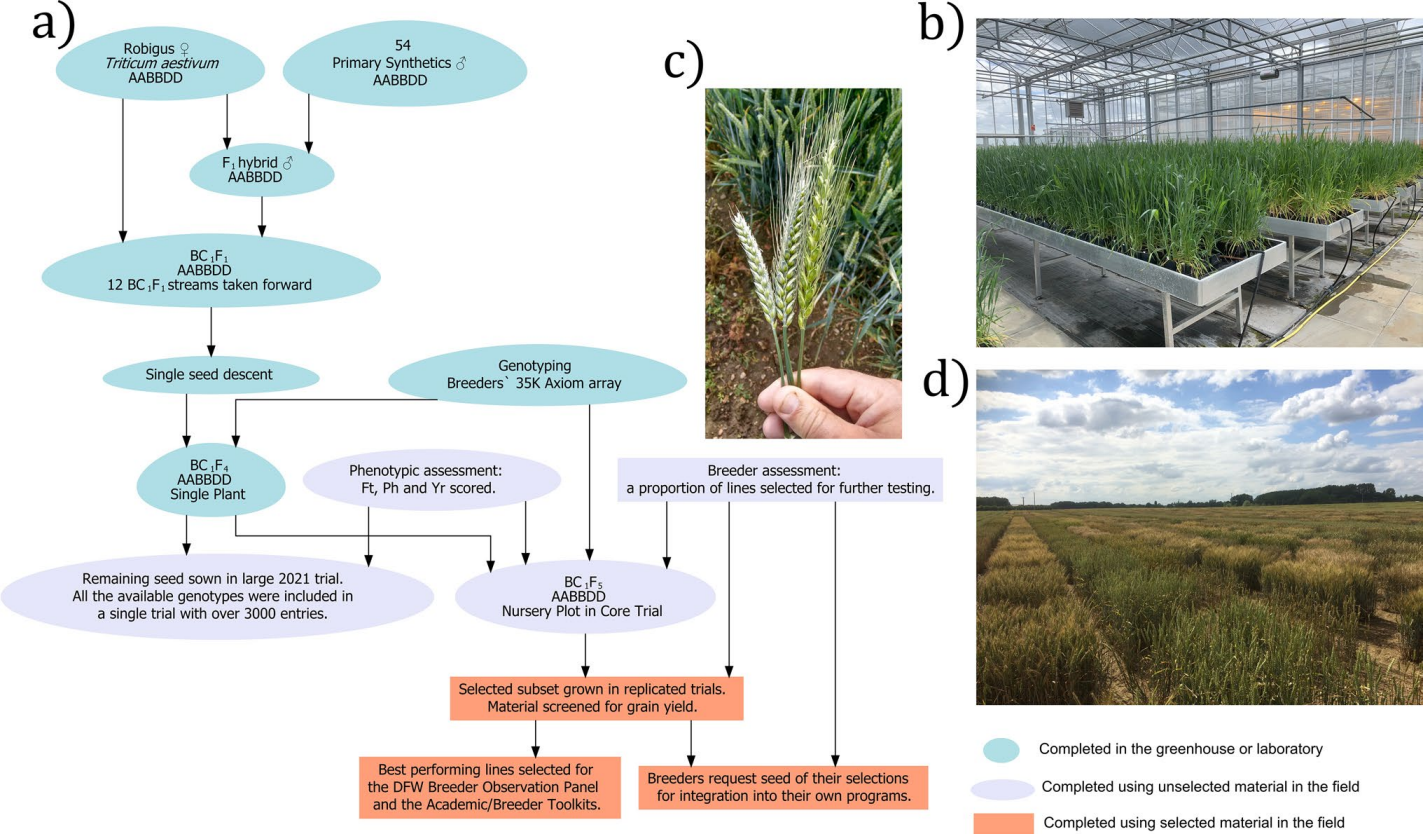
Development of the NIAB_WW_SHW_NAM population. **a** Workflow detailing how the NIAB_WW_SHW_NAM population was created, starting from the initial backcrossing of the primary SHW to ‘Robigus’, to field assessment and selection. **b** Generation advancement of NAM material at the back-cross 1 fourth filial (BC_1_F_4_) generation in the greenhouse. **c** Field-grown wheat ears showing from left to right: recurrent parent (UK wheat cultivar ‘Robigus’), Robigus / SHW BC_1_F_5_ progeny, and a primary SHW ear. **d** The ‘Core Nursery’ field trial from 2018

The elite UK winter wheat variety ‘Robigus’ was selected as the recurrent NAM parent. Released in 2000, this high-yielding, soft endosperm variety was widely grown across UK and elsewhere in North-Western Europe between 2003 and 2011 and is a key pedigree component in many recent and current UK varieties (Fradgley et al. 2019). For initial crossing, ‘Robigus’ was the maternal parent and primary SHW lines were pollen donors throughout. ‘Robigus’ × SHW first filial generation (F_1_) plants were crossed as pollen donors with ‘Robigus’ to produce back-cross 1 F_1_ (BC_1_F_1_) seeds. For the construction of each ‘Robigus’ × SHW nested population, 12 BC_1_F_1_ plants on average, were allowed to self-fertilize to produce BC_1_F_2_ seeds (Supplementary Table S2). Initial balanced nested populations of 96 BC_1_F_2_ genotypes per cross (eight BC_1_F_2_ from each of 12 BC_1_F_1_ streams) were rapidly advanced in modular seed trays through vernalisation and the glasshouse by Single Seed Descent (SSD) until BC_1_F_4_ seeds were harvested. A single BC_1_F_4_ individual (per genotype) was then sown in a 1L pot and grown in an unheated glasshouse under natural light from December to August to allow natural vernalisation, with resulting BC_1_F_5_ seed used for field planting. No phenotypic selection was carried out during population development. Figure 1 outlines the entire pipeline, from back-crossing to field testing of the NIAB_WW_SHW_NAM population.

### Field screening

#### Field trials

For logistical reasons, NAM development was staggered over several years, with different nested populations grown out as BC_1_F_5_ plots, denoted Core Nurseries, across several trial years. Autumn-sown trials were grown each year, typically from a sowing date in October and a harvest date the following August, from 2017–18 to 2021–22. Within the Core Nurseries, each BC_1_F_5_ genotype was grown in a single plant progeny 1.2 m^2^ observation plot consisting of six 1.2 m rows spaced 20 cm apart. Sequential plots were separated by short inter-plot gaps, with a much longer gap between each set of 20 plots; each sub-set of 20 plots is denoted as a ‘nursery drilling lid’. These trials were primarily used for initial field observations; there was no randomization or replication of genotypes, although ‘Robigus’ and primary SHW were repeated throughout each nursery. Information on each trial input and phenotypic assessment is shown in Table 1. Core Nurseries were generally grown with standard agrochemical inputs to control weeds, pests and diseases. However, the Core Nurseries planted in 2017 and 2018 (Core17 and Core18) had reduced fungicide applications to screen the material for resistance to natural yellow rust infection (*Yr*, also known as stripe rust, caused by the fungus *Puccinia striiformis* Westend f. sp. *tritici*).

**Table 1:** Trials used for field assessment of the NIAB_WW_SHW_NAM population

Planting year	Type of trial	Trial name	Fungicide application*	Nested populations in trial	NAM genotypes in trial	Traits assessed
2017	Core Nursery	Core17	Non-Standard	5	325	*Ft*, *Ph, Yr*
2018	Core Nursery	Core18	Non-Standard	19	1053	*Ft*, *Ph*, *Yr*
2019	Core Nursery	Core19	Standard	12	722	*Ft*, *Ph*
2020	Core Nursery	Core20	Standard	8	514	*Ft, Ph*
2021	Core Nursery	Core21	Standard	7	442	*Ft, Ph*
2021	Full Trial	Full21	Standard	51	2978	*Ft*, *Ph, Yr*

Phenotypic assessment was completed each harvest year for the agronomically important traits: flowering time (*Ft*), plant height (*Ph*) and yellow rust infection (*Yr*). The trials consisted of an annual series of Core Nursery experiments planted from 2017 to 2021 that each contained a proportion of the NAM population, as well as an experiment planted in 2021 which contained the majority of the NAM population (Full Trial)

*Non-standard refers to a reduced fungicide application to screen for resistance to infection

Most genotypes of the NIAB_WW_SHW_NAM population were assessed in the Full Trial (a single large field trial sown in 2021, termed hereafter ‘Full21’) (Supplementary Table S3). Here, BC_1_F_5_ genotypes were sown in an augmented field trial consisting of 2100 1.2 m^2^ plots. The experimental design was created in R (R Core Team 2022) with the package blocksdesign (Edmondson 2021). The Full21 trial included 2978 unreplicated BC_1_F_5_ genotypes of the NIAB_WW_SHW_NAM population (Supplementary Table S3), three replicates of 49 primary SHW parents, two primary SHW parents with a single replicate (due to seed availability) and three elite wheat check genotypes: ‘Robigus’, ‘KWS Santiago’ and ‘Theodore’, with 295, 285 and 444 replicates, respectively. ‘Robigus’ was included as the recurrent parent of the population; ‘KWS Santiago’ is often used by breeders as a check due to high yield and good yield stability, while ‘Theodore’ is visually distinctive and has a high untreated grain yield performance (AHDB 2022a). ‘Theodore’ was used to fill gaps where seed could not be found for a particular genotype. As a result of limited seed availability and the size of the population, each 6-row plot in the Full21 contained two different genotypes, one in drill coulters 1–3 and the second in coulters 4–6. To avoid replicated genotypes being allocated to the same plot, each plot was treated as a single experimental unit and blocksdesign (Edmondson 2021) was used to distribute the checks and replicated genotypes randomly across incomplete blocks (following Müller et al. 2010). This approach was implemented using the ‘design’ function of blocksdesign (Edmondson 2021), with three levels of blocking: block (consisting of 144 to 156 plots), trial rows and trial columns. Then, with each plot treated as two independent experimental units (half-plots), the non-replicated genotypes were randomized across the remaining units of the design. For the analysis, the intersection of each row or column within each block was treated as a nested sub-block. Across the complete trial, 24% of the entries were check varieties to adjust for field spatial variability. Genotypes from 51 of the 54 developed nested populations and their corresponding SHW parent were included in the trial with the remaining three populations (that were derived from the primary SHW: NIAB.SHW.008, NIAB.SHW.012 and NIAB.SHW.018), excluded due to limited seed availability; 58 genotypes per nested population were included on average (Supplementary Table S3).

#### Phenotypic assessment

Across the Core Nurseries and the Full Trial, three agronomically important traits were assessed: flowering time (*Ft*), yellow rust infection (*Yr*) and plant height (*Ph*). *Ft* was measured in days and counted from the drilling date to when 50% of the plot or half-plot (in the Full21 trial) had reached Zadoks growth stage 65 (Zadoks et al. 1974), namely half of the tillers in the plot were in anthesis. *Ph* was measured by a ruler and taken as the tallest point excluding awns of a single representative mature plant from each plot or half-plot. Natural infections of yellow rust in the trials were assessed using the standard methodology for scoring foliar disease in wheat variety trials (AHDB 2022b). The percentage of leaf area covered by yellow rust lesions was estimated for the top four leaves in the canopy, averaged across the whole plot (or half-plot). Scores were taken on June 1, 2018 (Core 17) and June 6, 2019 (Core 18), when plots typically ranged from GS55 to GS69. No assessments were made for the Core19, Core20 and Core21 trials as standard fungicide treatments gave good disease control. However, early yellow rust infection was observed in the Full21 trial and a score was made on April 25, 2022 (typically GS32) just prior to the first fungicide application.

#### Breeders’ selections

The Core17 to Core21 trials were visited by several commercial plant breeders, typically during July and early August. The purpose of these visits was to critically evaluate the previously unselected material and make selections of superior BC_1_F_5_ genotypes from a potential breeding value perspective, for further use in commercial breeding. Although details (which breeder selected which genotype) remained private, an overall summary was shared. To determine if it was possible to detect patterns in breeder selections, data on selections were summarized using plots created with the R packages waffle (Rudis and Gandy 2019) and ggplot2 (Wickham 2016).

#### Data analysis

Statistical analysis was conducted using R (R Core Team 2022) and RStudio (RStudio Team 2022). For computationally intensive jobs, analyzes were run on the ‘Crop Diversity High Performance Computing’ cluster (www.cropdiversity.ac.uk). For the traits (*Ft*, *Ph* and *Yr*) measured in the Full21 trial, Best Linear Unbiased Estimates (BLUEs) were estimated for each genotype using the following mixed linear model:

$${y}_{ijklmn}=\mu +{g}_{i}+{b}_{j}+{r}_{jk}+{c}_{jl}+{d}_{m}+{t}_{n}+{e}_{ijklmn}$$

where $y$ is the response of the $i$th genotype, in the $j$th block, in the $k$th row, the $l$th column, in the $n$th nursery drilling lid (subset of 20 plots) and measured by the $m$th scorer (the person who took the measurements). Furthermore, $\mu$ was the overall experimental mean, $g$ was the fixed genotypic effect of the $i$th genotype, $b$ was the random block effect of the $j$th block, $r$ and $c$ were the random row and column effect of the $k$th and $l$th row and column, respectively, nested within the $j$th block. Where appropriate, scorer effect was included via the random effect $d$ of the $m$th scorer. There was some physical separation of plots in the field based on which nursery lid they were drilled in, therefore, $t$ was included as the random effect of the $n$th nursery drilling lid. Lastly, $e$ was the residual error of each half-plot. For each trait, full models were fitted using the R package lme4 (Bates et al. 2015) and then reduced through backward elimination of the random terms implemented via the ‘step’ function of the R package lmerTest (Kuznetsova et al. 2017).

In the Full21 trial, broad-sense heritability (*H*^2^) was estimated as ${H}^{2}={V}_{g}/{V}_{p}$, where ${V}_{g}$ was the variance associated with genotype treated as a random factor in the final model for each trait. Phenotypic variance (${V}_{p}$) was the sum of ${V}_{g}$ + ${V}_{e}$, where ${V}_{e}$ was the residual error variance from each model. Implemented by the R functions ‘cor’ and ‘cor.test’ (R Core Team 2022), a Pearson’s correlation test was used to test the correlation of *Ft*, *Ph* and *Yr* across trials for all the genotypes included in the study. For the three traits, two sample t-tests were completed using the R function ‘t.test’ to test for significant differences between the means of the genotypes selected by ≥ 50% of breeders in each Core Nursery compared to genotypes not selected. Using the same approach, t-tests were used to test for significant differences between genotypes with different homozygous alleles at a QTL linked to *Yr* found in each of the trials. Phenotype distributions and correlations were plotted using the R packages ggplot2 (Wickham 2016) and ggcorrplot (Kassambara 2022).

### Genotype analysis

#### DNA extraction and genotyping

DNA was extracted from seedling leaf tissue of the NIAB_WW_SHW_NAM population and parental lines (recurrent parent ‘Robigus’ and primary SHW), following an adapted protocol from Fulton et al. (1995). The extracted DNA was genotyped using the Axiom® 35K Wheat Breeders’ single nucleotide polymorphism (SNP) array (ThermoFisher Scientific) at Bristol University (Allen et al. 2017). The initial genotyping included 3282 genotypes from either BC_1_F_4_ or BC_1_F_5_ nested populations, 161 replicates of primary SHW and 32 ‘Robigus’ replicates (used as ‘batch’ controls between genotyping events).

Genetic marker calling was completed using the Axiom Analysis Suite (AAS) software (version 5.1, www.thermofisher.com). Within the software, thresholds for the quality control (QC) of the genotype samples included an inbred penalty of 4, a dish quality control of 0.82 and a QC call rate of 96%. There were six replicates of primary SHW parents that exhibited slightly below threshold QC call rates, these were still advanced to the next stage of the analysis as they were parental lines. SNP genotyping was completed with an inbred penalty of 4 and an initial call rate cut-off of 96%, although all genetic markers above a call rate cut-off of 94% were manually inspected and markers were curated and kept where clustering was still appropriate (i.e., clear separation between at least two genotype classes: AA, AB and BB). After the initial filtering, 14,425 SNP markers and 2961 genotypes progressed to the next stages of QC. Of these markers, 7690 were initially classified as ‘NoMinorHom’ (no minor homozygote alleles observed) in AAS; these were all manually inspected and were recalled if: three distinct clusters were visible (AA, AB, BB) or if there was still clear separation between two clusters, the heterozygous clusters were assumed to represent the missing homozygous class.

#### Genotype data QC, imputation, and genetic marker positioning

The downstream analysis was conducted using R Core Team (2022) and RStudio Team (2022). Genetic markers which exceeded one or more of the following QC criteria were removed: over 10% heterozygote calls, over 10% missing data, and if fewer than 15 genotypes were homozygous for the minor allele. Genotypes with over 10% heterozygous marker calls were also removed. Missing data per genotype was low (typically < 6%) and no genotypes were excluded due to missing data. Principal Coordinate Analysis (PCoA), implemented by the R package ape (Paradis and Schliep 2019), was used to identify genotypes within each nested population that were outliers. Pearson’s correlation coefficients were calculated between all pairs of genotypes and plotted. Outliers that suggested a genotype pair were too similar, compared to the overall distribution of all pairs, were investigated and erroneous genotypes were removed. Within nested populations, monomorphic markers (between ‘Robigus’ and each primary SHW) were used to find further outlying genotypes and these were removed from the analysis. Implemented through the R package missForest (Stekhoven 2022), missing genotypic data was imputed using Random Forest with 200 trees grown in each forest. Replicates of parental genotypes were removed and after these stages of QC there were 11,227 genetic markers and 2726 genotypes remaining in the dataset.

Physical map positions for 10,128 of these SNPs, based on the wheat reference genome assembly of cv. ‘Chinese Spring’ (IWGSC 2018, RefSeq v1.0), were downloaded from ‘CerealsDB’ (Wilkinson et al. 2012). To obtain physical positions for more of the remaining unmapped genetic markers, the SNP probe sequences of the 35 K Wheat Breeders’ array were downloaded from ‘CerealsDB’. With default parameters, these DNA sequences were queried through BLAST + (version 2.12.0, Camacho et al. 2009) against the IWGSC RefSeq v1.0 wheat assembly. For each BLAST hit, the SNP position was taken as the median base pair between the start and stop of the alignment. Physical positions for a further 946 markers were identified by comparing correlation (*R*^2^) with markers that had known positions and taking the nearest BLAST hit of the unmapped marker on the same chromosome to the already positioned marker (if present). As an additional step to improve marker positioning, each marker was placed in a bin with markers that were in high linkage disequilibrium (LD) with each other (*R*^2^ > 0.5). If the marker was located on a different chromosome to the majority of markers in the bin and that marker had a BLAST hit on the most represented chromosome in the bin, it was repositioned using that BLAST hit. If no consensus chromosome could be identified, the chromosome of the marker in highest LD was used as the guide. This approach was run twice, and a total of 2852 markers were repositioned. Heatmaps showing LD on each chromosome were inspected to visualize the improvement of the map reordering and the plots are shown in Supplementary Figure S1. All heatmaps were plotted using the R package LDheatmap (Shin et al. 2006).

One nested population (derived from NIAB.SHW.091) in the final dataset was excluded due to possible cross contamination identified in the QC process. The final dataset contained 11,051 mapped markers for 2637 NAM genotypes, 54 primary SHW parents and the recurrent parent ‘Robigus’.

#### Population structure

To understand the relationship between the NIAB_WW_SHW_NAM population and parents, a PCoA was performed on SNP data that was ‘skimmed’ to remove a marker in each pair of markers with an absolute Pearson’s correlation (*r*) ≥ 0.8. The PCoA was also completed using ‘skimmed’ D sub-genome genetic markers of the primary SHW donors and ‘Robigus’. Distinct clusters identified by plotting the first two principal coordinates were assigned to known lineages of *Ae. tauschii* using overlapping genotypes between the present study and Gaurav et al. (2022). The latitudes and longitudes of the passport data location of the *Ae. tauschii* accessions used to make the primary synthetics were plotted on a map to show geographical distribution, conducted using the R packages rnaturalearth (Massicotte and South 2017) and sf (Pebesma 2018). Plots showing population structure and geographical distribution were created using the R package ggplot2 (Wickham 2016).

#### Association mapping

Of the NIAB_WW_SHW_NAM 2637 genotypes with genetic marker data, there were phenotypic data available for 2445 genotypes from 51 nested populations; three of the nested populations were not phenotyped. Association mapping used an additive Q + K model implemented using the R package GWASpoly (Rosyara et al. 2016), where population structure (Q) was the nested population number included as a covariate and kinship (K) was a marker estimated relationship matrix calculated using the GWASpoly function ‘set.K’ with leave-one-chromosome-out set to false. Genotype number varied across experiment (Table 1), so for each trial, genetic markers that had less than 15 genotypes homozygous for the minor allele were removed and the remaining markers were ‘skimmed’ to remove non-unique markers, i.e., where a marker pair had an absolute *r* = 1. For each trial, marker sets for kinship estimation were formed using an additional skim of *r* = 0.80, to reduce areas of high genetic marker LD biasing the estimates of kinship. The final model thresholds were determined by testing different levels of marker skimming and types of population covariates using the Full21 trial that contained data from most of the population (an example for the trait *Ft* is shown in Supplementary Figures S2 and S3). To ensure that appropriate corrections for relatedness between genotypes had been made, histograms of observed *P*-value distribution and quantile–quantile plots were inspected, and genomic inflation factors were estimated (Devlin and Roeder 1999).

Using the GWASpoly function ‘set.threshold’, significance thresholds were calculated at a corrected *P* = 0.05, by estimating effective markers through accounting for LD between SNPs and then using a Bonferroni-type correction.With the genetic data used in mapping for the Full21 trial and the GWASpoly function ‘LD.plot’, pairwise LD between SNPs were plotted against physical distance to examine LD decay to a critical value of *R*^2^ = 0.2. Initially, candidate QTLs were taken as the peak marker within the determined window (Mb) of LD decay (*R*^2^ = 0.2). Using a criterion of marker −log_10_(*P*) score, allele effect direction (using ‘Robigus’ as the reference) and physical marker location, candidate QTLs were visually inspected within and between trials, and QTLs were named and neighboring QTL merged where appropriate. QTL intervals were visually defined as the first and last significant flanking genetic markers surrounding each peak. For each trial, the GWASpoly function ‘fit.QTL’ was used to combine the final set of candidate QTLs for each trait into a multiple QTL model to estimate phenotypic variance explained by each QTL.

### Material and data availability

Information about the availability of seed for the genotypes of the NIAB_WW_SHW_NAM population is accessible from: www.niab.com/research/agricultural-crop-research/resources/niab-wheat-nested-association-mapping-nam-panels. The population genetic marker and field phenotype data is available from: https://niab.github.io/niab-dfw-wp3/.

## Results

### The NIAB_WW_SHW_NAM population

The NIAB_WW_SHW_NAM resource had 3241 genotypes from 54 BC_1_F_5_ nested populations (average population size = 60), based on the greatest number of lines either genotyped or phenotyped per nested population (Supplementary Table S2). The full parentage of the primary SHW genotypes and information on the origin of the parents is shown in Supplementary Table S1. Across the field experiments, 3056 genotypes were phenotyped from 51 nested populations. The quality-controlled genotype data comprises of 2637 genotypes from 54 nested-populations. Both genetic marker and phenotype data were available for 2445 NIAB_WW_SHW_NAM genotypes, from 51 nested populations. Supplementary Table S2 details the numbers of genotypes phenotyped and genotyped in each nested population.

**Fig. 2:**
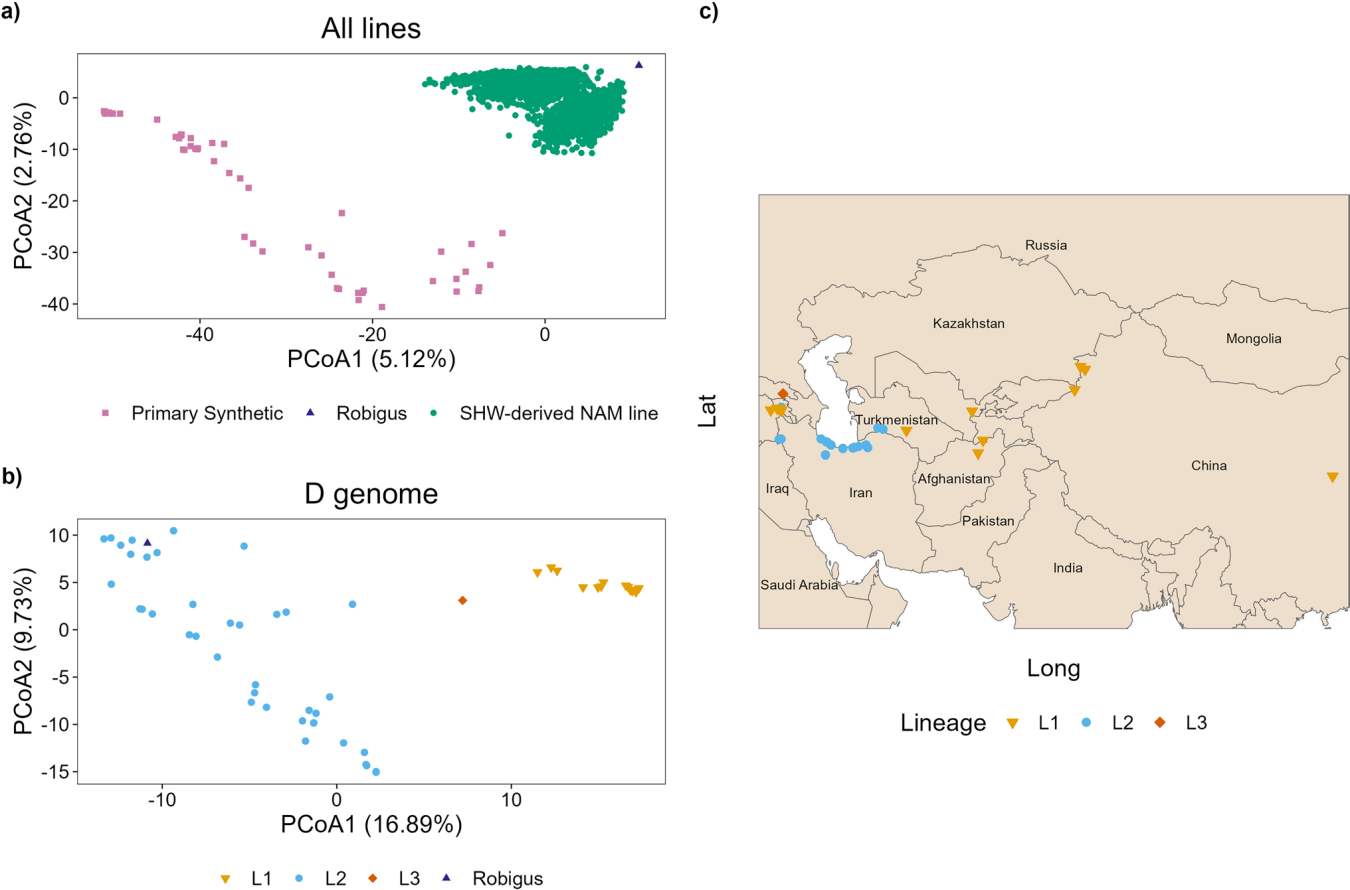
The NIAB_WW_SHW_NAM population is based on genetically diverse founders originating from a wide geographical area. Principal coordinate analysis (PCoA) of the first two principal coordinates for the NIAB_WW_SHW_NAM population genotypes and their parents (**a**) and the D sub-genome genetic markers for the primary synthetic hexaploid wheat donors and ‘Robigus’ (**b**), split into the three *Ae. tauschii* lineages (L1, L2, L3). The lineages of *Ae. tauschii* were assigned to the distinct clusters using overlapping genotypes between the present study and Gaurav et al. (2022). **c** A map showing the passport data location of 43 *Ae. tauschii* (diploid, D genome) accessions captured in the primary SHW (hexaploid, ABD genome)

The population structure of the NIAB_WW_SHW_NAM population and parental genotypes is shown in Fig. 2. The first PCoA shows the genotypes of the population (green) clustered between their parents ‘Robigus’ (recurrent parent, dark blue) and the primary SHW (donor parent, pink) (Fig. 2a). Genotypes clustered closer to ‘Robigus’, as would be expected for BC_1_ derivatives. For the NIAB_WW_SHW_NAM genotypes and parental lines, the first two principal coordinates accounted for 7.88% of the variation (PCoA1 = 5.12%, PCoA2 = 2.76%, Fig. 2a). A second PCoA, using just the D sub-genome genetic markers of the population parents (primary SHW and ‘Robigus’), shows distinct clusters (Fig. 2b). Using overlapping *Ae. tauschii* donors between the present study and Gaurav et al. (2022), these were assigned to lineages 1, 2 and 3 (L1, L2 and L3), the three previously categorized lineages of *Ae. tauschii.* The genotypes covered all three lineages, with ‘Robigus’ clustering within L2 (Fig. 2b). Only one primary SHW (NIAB.SHW.092) was found within the putative L3.

The geographic collection site locations (based on passport data) of the *Ae. tauschii* donors captured in the primary SHW parental lines are shown in Fig. 2c, colored by lineage (Fig. 2b). This shows that L1 accessions are spread out across the geographical distribution of *Ae. tauschii* and separated by the Caspian Sea. The majority of the L2 accessions are grouped south of the Caspian Sea in Iran. The single L3 accession was sampled from Georgia (Fig. 2c).

### Phenotypic characterization

**Fig. 3:**
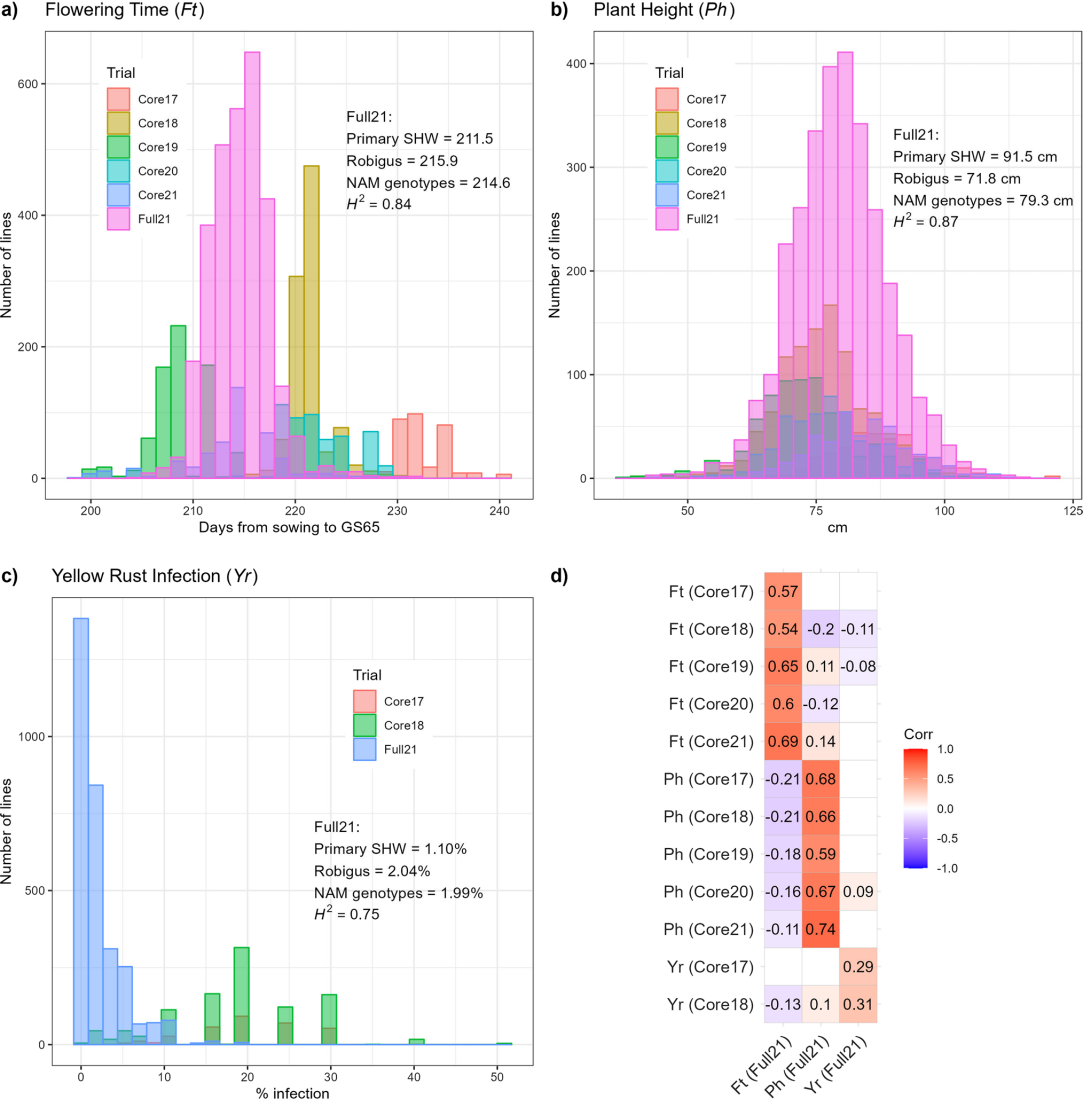
Histograms of phenotype distributions for the three traits assessed in the NIAB_WW_SHW_NAM population: flowering time (*Ft*; **a**), plant height (*Ph*; **b**), yellow rust infection (*Yr*; **c**), from the Core17 to Core21 and Full21 trials. From the Full21 trial, the mean of all the Best Linear Unbiased Estimates (BLUEs) of the NAM population genotypes and the mean of all the BLUEs of the primary SHW is shown with the BLUE of the recurrent parent ‘Robigus’ for comparison. **d** Pearson’s correlation coefficient (*r*) heatmap for the three traits using data from genotypes present in the Full21 trial and each Core Nursery in which each blank square represents an insignificant *r* (*P* > 0.05)

Across five successive Core Nurseries (Core17 to Core21) and the Full Trial (Full21), the NIAB_WW_SHW_NAM population was assessed for three traits: flowering time (*Ft*), plant height (*Ph*) and yellow rust infection (*Yr*)*.* Results and statistics from the trials are summarized in Fig. 3. As the Full21 trial was replicated and randomized, Best Linear Unbiased Estimates (BLUEs) and broad-sense heritability (*H*^2^) were calculated. High *H*^2^ was observed for all traits (*Ft* = 0.84, *Ph* = 0.87 and *Yr* = 0.75). For the Full21 trial, the BLUEs for all the population genotypes were averaged and compared to the average of all the SHW donors and the BLUE of ‘Robigus’ (Fig. 3a–c). For all traits in this trial, the means of the population genotypes were inside the range of the BLUE for ‘Robigus’ and the means of the BLUEs for the SHW donors. However, there were individual population genotypes outside of the parental ranges; transgressive segregation was observed for all traits in the Full21 trial (Fig. 3a–c). In the Full21 trial, the SHW parents were early flowering (*Ft*), with a mean of 211.5 days from sowing to GS65, compared to ‘Robigus’ (215.9 days). In contrast, the SHW parents were taller with a mean *Ph* of 91.5 cm compared to the recurrent parent ‘Robigus’ (71.8 cm). The SHW parents showed lower yellow rust infection in the Full21 trial (*Yr* = 1.1%) compared to ‘Robigus’ (*Yr* = 2.0%). For all three traits the means of the genotypes were closer to the BLUE of the recurrent parent ‘Robigus’ (Fig. 3a–c).

The trait distributions changed across both year and trial (Fig. 3a–c). Across years, for example, *Ft* in the Core17 trial was later than in all other trials (Fig. 3a), while *Ph* was generally higher in the Full21 trial (Fig. 3b). In those trials that had reduced fungicide treatment (Core17 and Core18), disease pressure was higher than the Full21 trial which had a standard fungicide application (Fig. 3c). As shown in Fig. 3d, regardless of the differences in distribution, *Yr* in the Full21 trial was weakly positively correlated with *Yr* in the Core17 (*r* = 0.29, *P* < 0.001) and Core18 nurseries (*r* = 0.31, *P* < 0.001). The same trend was observed for *Ph* and *Ft*, although the correlations were higher. For *Ph* the correlation of the nurseries with the Full21 trial ranged from *r* = 0.59 to 0.74, while for *Ft* the correlation ranged from *r* = 0.54 to 0.69. Across trials, the Core21 nursery and Full21 trial were grown in the same year and had the highest correlation for *Ft* (*r* = 0.69, *P* < 0.001) and *Ph* (*r* = 0.74, *P* < 0.001). Across traits, there were weaker correlations observed between the Full21 trial and the Core Nurseries. For example, *Ft* in the Full21 trial was weakly negatively correlated with *Ph* in Core Nurseries, with correlations ranging from *r* =  − 0.11 to − 0.21 (Fig. 3d).

### Breeders’ selections

Across the five Core Nurseries, a relatively high percentage of lines per trial were selected by breeders for inclusion into their breeding pipelines (≥ 21% per year; Supplementary Table S4). The numbers of breeders visiting increased from the Core17 to Core21 trial, with eight breeders making selections on the material in the Core21 trial. In parallel, a higher proportion of genotypes were selected in the later Core Nurseries (Supplementary Table S4). The differences between trait distributions for selected versus non-selected genotypes are shown in Supplementary Figure S4. The Core17 and Core18 nurseries had reduced fungicide application and there were significant differences for the means of selected versus non-selected genotypes (Supplementary Figure S4). For example, the *Yr* means were 16.9% versus 21.4% in 2017 and 7.1% versus 20.2% in 2018, for selected and non-selected genotypes, respectively. Less consistent directional selection was observed for the traits *Ft* and *Ph* (Supplementary Figure S4).

**Fig. 4:**
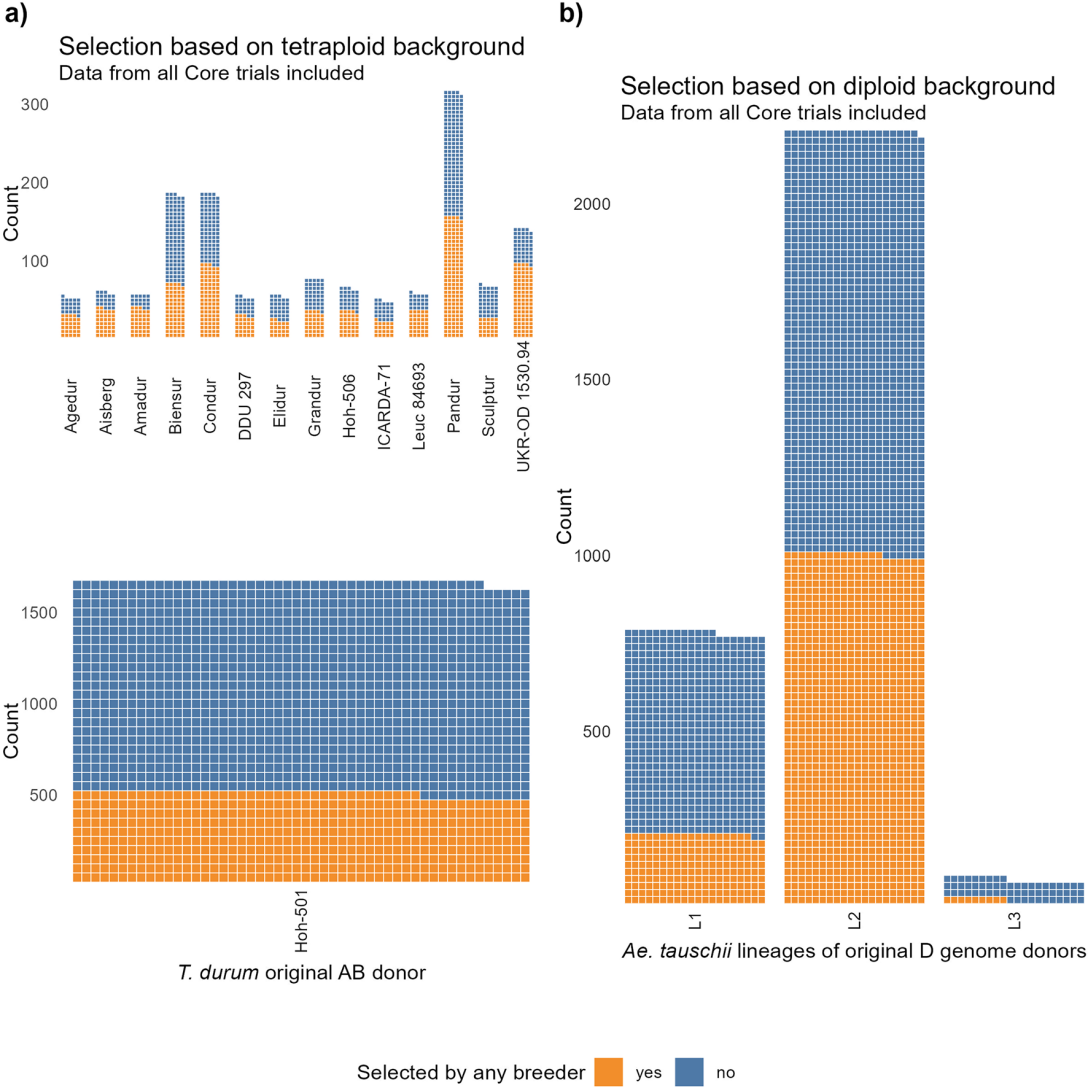
Selection patterns are shown in the tetraploid (**a**) and diploid lineage (**b**) backgrounds for the population genotypes represented by each square. A genotype is shown as ‘selected’ if it was selected by any breeder across the Core Nurseries

Next we considered the consequence of breeder selection, based on which *T. durum* (AB) and *Ae. tauschii* (D) SHW sub-genome donors were present in the selected versus non-selected genotypes. It should be noted that the donor contributions across the NAM were not balanced (particularly with the *T. durum* donors, Supplementary Table S2). Additionally, the inclusion of donor backgrounds within each Core Nursery was not randomized, as nested populations were developed as cohorts. Therefore, only visual trends are commented on. The visual patterns of the breeders’ selection versus donor backgrounds are shown in Fig. 4a, b, where ‘selected genotypes’ indicate if any breeder selected that genotype within any of the Core Nursery trials. *T. durum* ‘Hoh-501’ was the most common tetraploid background used in the generation of primary SHW (Fig. 4a), and 29.7% of the 1645 BC_1_F_5_ genotypes with ‘Hoh-501’ as a donor in their primary SHW parent were selected by breeders. Aside from the ‘Sculptur’ and ‘Biensur’ *T. durum* backgrounds (where selection was 37.9% and 37.7%, respectively), selection was greater than 40% across all other *T. durum* backgrounds (Fig. 4a). Notably, 69.1% and 67.6% of genotypes with ‘Amadur’ and ‘UKR-OD 1530.94’, respectively, were selected. Selections based on the diploid background are shown in Fig. 4b. *Ae. tauschii* donors from the L2 lineage were the most used source of D sub-genome for SHW creation (Supplementary Table S1-S2) and were also most enriched in the selected versus unselected genotypes (45.2% selected). Selection was considerably lower in genotypes derived from SHW with *Ae. tauschii* lineage L1 donors, with 25.6% of 773 selected. Of the 69 L3 genotypes available for selection, only 13.0% were selected by the breeders.

### Genetic mapping

The three assessed traits (*Yr*, *Ft* and *Ph*) were used for QTL mapping. LD was determined to decay (*R*^2^ = 0.2) at a window of 18 Mb (Supplementary Figure S5). After the marker scores (−log_10_(*P*)) were computed in each GWAS run, QTLs were initially called as the most significant SNP across a 18 Mb window. These QTLs were named, and where appropriate combined, using visual inspection of marker scores (−log_10_(*P*)), SNP effects and taking the physical location of markers into account. All six trials were used and QTLs that were found in at least two trials are summarized in Table 2. In total, 27 QTLs were found across all trials and traits (Supplementary Table S5), of which eight were found in at least two trials (Table 2). Manhattan plots for all three traits across all trials are shown in Supplementary Figures S6, S7 and S8.

**Table 2:** QTLs found across multiple trials using the NIAB_WW_SHW_NAM population for flowering time (*Ft*), plant height (*Ph*) and yellow rust infection (*Yr*)

QTL	Trait	Trial	*n*	Sig. threshold	Genetic marker	Chr	Peak Position (Mb)	–log_10_(*P*)	Alt. allele effect	Var. (%)
*QFt.niab-2B.1*	*Ft*	Core19	596	4.86	AX.94970315	2B	26.6	6.34	− 0.96	0.84
*QFt.niab-2B.1*	*Ft*	Full21	2389	4.97	AX.95082190	2B	58.1	6.25	− 0.99	1.10
*QFt.niab-2D.1*	*Ft*	Core19	596	4.86	AX.94779177	2D	14.9	5.33	− 0.86	0.04
*QFt.niab-2D.1*	*Ft*	Core21	277	4.66	AX.94603120	2D	33.4	10.74	− 1.77	10.75
*QFt.niab-4A.1*	*Ft*	Core18	852	4.66	AX.94552332	4A	735.8	10.09	− 0.68	0.18
*QFt.niab-4A.1*	*Ft*	Full21	2389	4.97	AX.95165912	4A	681.4	10.31	− 0.84	0.86
*QFt.niab-7D.1*	*Ft*	Core18	852	4.66	AX.95229555	7D	17.8	8.50	− 0.62	0.03
*QFt.niab-7D.1*	*Ft*	Core20	457	4.66	AX.94929727	7D	61.2	5.87	− 1.06	5.07
*QFt.niab-7D.1*	*Ft*	Full21	2389	4.97	AX.94688897	7D	32.0	5.33	− 0.59	< 0.01
*QPh.niab-5A.2*	*Ph*	Core18	852	4.66	AX.94462177	5A	527.9	8.52	2.25	0.34
*QPh.niab-5A.2*	*Ph*	Full21	2389	4.97	AX.94462177	5A	527.9	5.36	1.50	1.07
*QPh.niab-6A.1*	*Ph*	Core17	253	4.46	AX.94474129	6A	114.2	6.18	3.61	0.87
*QPh.niab-6A.1*	*Ph*	Core18	852	4.66	AX.95630086	6A	230.7	12.63	3.18	3.28
*QPh.niab-6A.1*	*Ph*	Core21	277	4.66	AX.95630086	6A	230.7	6.73	4.25	9.81
*QPh.niab-6A.1*	*Ph*	Full21	2389	4.97	AX.95159326	6A	405.4	19.10	3.06	2.45
*QPh.niab-6D.1*	*Ph*	Core17	253	4.46	AX.94940605	6D	84.3	6.06	3.43	0.99
*QPh.niab-6D.1*	*Ph*	Core18	852	4.66	AX.94940605	6D	84.3	6.71	2.01	< 0.01
*QPh.niab-6D.1*	*Ph*	Full21	2389	4.97	AX.94940605	6D	84.3	6.64	1.58	< 0.01
*QYr.niab-4D.1*	*Yr*	Core17	253	4.46	AX.94546744	4D	1.4	4.90	− 2.25	7.89
*QYr.niab-4D.1*	*Yr*	Core18	852	4.66	AX.94546744	4D	1.4	14.27	− 3.93	7.76
*QYr.niab-4D.1*	*Yr*	Full21	2389	4.97	AX.94546744	4D	1.4	13.82	− 0.78	2.98

The chromosome (Chr.) and physical position (measured in Mb) for each peak marker of each QTL are listed. ‘−log_10_(*P*)’ represents the −log_10_(*P*-value) for each QTL; ‘Var.’ shows percentage variation explained by each QTL. Significance thresholds (Sig. threshold) were estimated with a Bonferroni-type correction that uses effective markers based on linkage disequilibrium for a corrected *P* = 0.05. The ‘Alt. allele effect’ represents the dosage of the alternative SHW allele (where ‘Robigus’ was the reference) and ‘*n*’ was the number of genotypes used in the genetic mapping for each trial

**Fig. 5:**
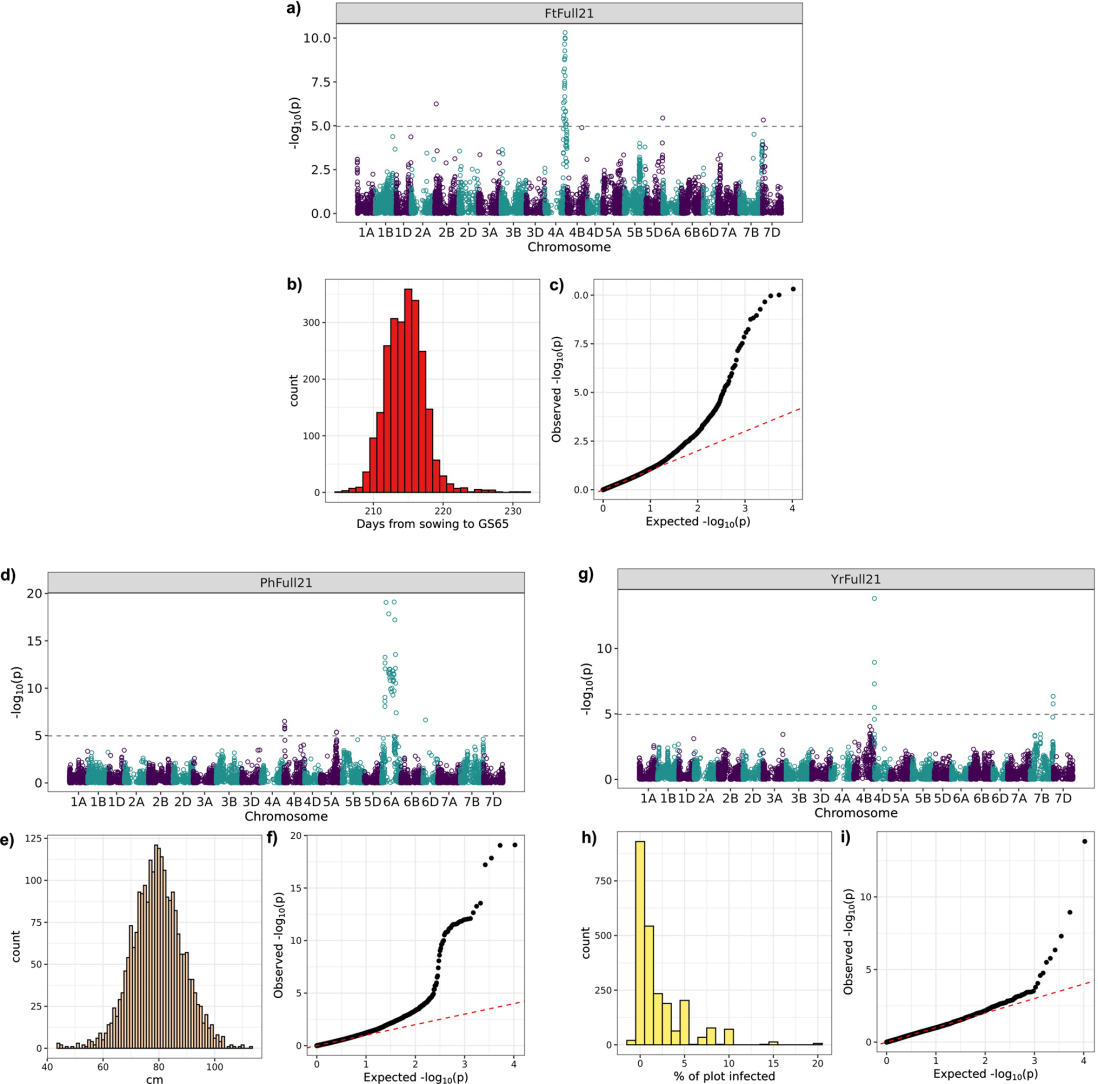
QTL mapping results from the Full21 trial for the three traits: flowering time (*Ft*; **a**), (**b**) and (**c**)), plant height (*Ph*; **d**), (**e**) and (**f**)) and yellow rust infection (*Yr*; **g**), (**h**) and (**i**)). For each trait, a Manhattan plot is shown, with genetic markers ordered based on physical map position (**a**), (**d**) and (**g**)). Bonferroni-like corrections using effective markers determined by linkage disequilibrium were used to estimate the significance thresholds for a corrected *P* = 0.05. The phenotype distributions of all genotypes used in the mapping (**b**), (**e**) and (**h**)) and the quantile–quantile plot for each QTL scan (**c**), and (**f**) and (**i**)) were also plotted for each trait

Across the trials, 18 QTLs for *Ft,* on 13 chromosomes, were found in total (Supplementary Table S5). Of these, four QTLs were identified across multiple trials, with the alternative SHW allele contributing to earlier flowering in each case (Table 2). The most significant of these four was *QFt.niab-2D.1*_*wSHWnam*_ on chromosome 2D in the Core21 trial (−log_10_(*P*) = 10.7), which explained 10.8% of the phenotypic variation for *Ft*. This was also found in the Core19 trial (−log_10_(*P*) = 5.3) with a 18.5 Mb position change in the peak marker. *QFt.niab-2D.1*_*wSHWnam*_ was the only QTL found across multiple trials that was not identified in the Full21 trial, typically the other QTLs were found in the Full21 trial and at least one Core Nursery (Table 2). The most significant hit for *Ft* in the Full21 trial was *QFt.niab-4A.1*_*wSHWnam*_ (−log_10_(*P*) = 10.31), located on chromosome 4A at 681.4 Mb (Fig. 5), where the QTL interval ranged from 627.8 to 726.3 Mb (Supplementary Table S5). This QTL was identified in the Core18 trial across a similar interval on 4A (625.4 Mb to 744.5 Mb), although the peak marker position shifted to 735.8 Mb. *QFt.niab-7D.1*_*wSHWnam*_ was found at the start of chromosome 7D in three trials (including the Full21 trial, Fig. 5) and spanned a 59.3 Mb QTL interval across the trials. *QFt.niab-7D.1*_*wSHWnam*_ explained a low percentage (< 1%) of the phenotypic variation in the Core18 and Full21 trial, but explained more of the variation in the Core20 trial (5.1%). *QFt.niab-2B.1*_*wSHWnam*_*,* located on chromosome 2B at a similar physical position to *QFt.niab-2D.1*_*wSHWnam*_ on 2D*,* was significant in the Full21 and Core19 trials where the peak marker was found at 58.1 and 26.6 Mb, respectively (Table 2).

For *Ph* seven unique QTLs were found in total (Supplementary Table S5) including three QTLs detected across multiple trials (Table 2). *QPh.niab-6A.1*_*wSHWnam*_ was a highly significant QTL detected across four trials including the Full21 trial, with a −log_10_(*P*) score of 19.1 (Fig. 5). This QTL was found close to the pericentromeric region of chromosome 6A, with the peak markers from 114.2 to 405.4 Mb (Table 2), and a large QTL interval which spanned from 50.7 to 495.7 Mb (Supplementary Table S5). For six out of the seven *Ph* QTLs detected, including *QPh.niab-6A.1*_*wSHWnam*_, the alternative SHW allele increased height (Supplementary Table S5). *QPh.niab-6D.1*_*wSHWnam*_ was found at the start of chromosome 6D in three trials, including the Full21 trial, with the same peak marker (AX.94940605) located at 84.3 Mb (Fig. 5). This QTL explained a low percentage of variation in each trial (< 1%), whereas *QPh.niab-6A.1*_*wSHWnam*_ explained between 0.9 and 9.8% (Table 2). The third replicated *Ph* QTL was *QPh.niab-5A.2*_*wSHWnam*_, with the peak marker AX.94462177 located at 527.9 Mb on chromosome 5A. This QTL was significant in the Core18 and Full21 trial (Fig. 5). In the Core17 and Core19 trial the peak on 5A was just below the significance threshold (Supplementary Figure S7) and in the Core20 trial a close QTL was detected at 467.0 Mb (*QPh.niab-5A.1*_*wSHWnam*_, Supplementary Table S5).

Three trials were used for scoring *Yr*. Trials Core17 and Core18 had reduced fungicide application, with *Yr* scored in early June, while trial Full21 received a *Yr* assessment in late April, just prior to the first application of a standard fungicide regime. In total, two *Yr* QTLs were found, of which only one was identified across all three trials (*QYr.niab-4D.1*_*wSHWnam*_, Table 2). This QTL had the same peak marker in all three trials (AX.94546744) located at 1.4 Mb at the start of chromosome 4D and was highly significant in the Full21 and Core18 trials (−log_10_(*P*) = 13.8 and 14.3, respectively). Out of the 54 primary SHW parents, 37 were homozygous for the allele linked to increased *Yr* resistance at the peak SNP for *QYr.niab-4D.1wSHWnam* (AX.94546744). In the Full21 trial, 34 primary SHW lines with this resistance allele had an average *Yr* score significantly lower than 16 primary SHW lines with the same allele as ‘Robigus’, which showed increased susceptibility to *Yr* (Supplementary Figure S9). The significance of *QYr.niab-4D.1*_*wSHWnam*_ was lower in the Core17 trial (−log_10_(*P*) = 4.9), this trial had far fewer genotypes than in Core18 and Full21, which may have reduced the statistical power. The QTL interval on 4D across the three trials was small and ranged from 1.3 to 3.6 Mb (Supplementary Table S5). The QTL explained between 3.0 to 7.9% of the *Yr* phenotypic variation (Table 2). A second QTL, *QYr.niab-7B.1*_*wSHWnam*_ QTL, was found only in the Full21 trial (Supplementary Table S5). For both QTLs, the alternative SHW allele conferred a lower *Yr* score, indicating increased yellow rust resistance.

To see if any other QTLs could be identified, GWAS scans were repeated for the Full21 trial using major hits as covariates (Supplementary Figure S10). The major hits were taken as the peak marker for QTLs found in both the Full21 trial and at least one of the Core Nurseries (*Ft*: *QFt.niab-2B.1*_*wSHWnam*_, *QFt.niab-4A.1*_*wSHWnam*_ and *QFt.niab-7D.1*_*wSHWnam*_; *Ph*: *QPh.niab-5A.2*_*wSHWnam*_, *QPh.niab-6A.1*_*wSHWnam*_ and *QPh.niab-6D.1*_*wSHWnam*_; *Yr*: *QYr.niab-4D.1*_*wSHWnam*_). For *Ft*, *QFt.niab-5D.1*_*wSHWnam*_ was the only QTL observed above the significance threshold and no new QTLs were found. Similarly, for *Ph* only *QPh.niab-4B.1*_*wSHWnam*_ was significant and no new QTLs were found. For *Yr, QYr.niab-7B.1*_*wSHWnam*_ was significant, together with a marginal additional hit on chromosome 4B at 528.0 Mb (−log_10_(*P*) = 4.99), for which the ‘Robigus’ allele increased *Yr* resistance. In summary, aside from the additional marginal hit found for *Yr*, covariate analysis detected no additional QTLs.

## Discussion

### The NIAB_WW_SHW_NAM population

The NIAB_WW_SHW_NAM population, consisting of 3241 genotypes created from 54 primary SHW, represents a large, genetically diverse resource for accessing novel D sub-genome variation from the wheat progenitor *Ae. tauschii*. To aid rapid exploitation via pre-breeding, the population makes this diversity available in the adapted UK elite wheat background ‘Robigus’ together with associated genome-wide high density SNP genotype data. The population is the largest publicly available resource of genotyped SHW-derived germplasm in a North European elite winter wheat background.

The 47 D sub-genome donors of the NIAB_WW_SHW_NAM population represent the three known *Ae. tauschii* lineages. This includes 14 primary SHW formed from Lineage 1 (L1) donors, which are considered to be under-utilized in modern wheat breeding (Singh et al. 2019). Lineage 2 (L2) is thought to include the ancestral donor of wheat (Wang et al. 2013), with a geographical distribution around the south of the Caspian Sea, consistent with the accessions used here. More recent evidence supports the contribution of the putative ‘Lineage 3’ (L3) to the modern wheat D sub-genome; Gaurav et al. (2022) attributed genetic signatures from L2 and the L3 in modern wheat. One primary SHW (NIAB.SHW.092) captures the D sub-genome of the donor *Ae. tauschii* line ‘Ent 272’, which has previously been assigned to the putative L3 by Gaurav et al. (2022). In the formation of the NIAB_WW_SHW_NAM population, the highest proportion of *Ae. tauschii* donors came from L2, with these accessions contributing to 39 of the primary SHW used as population donors.

### Flowering time (Ft)

Incorporating novel chromosomal introgressions from progenitor species into modern elite genomes risks disrupting desirable allelic combinations, assembled over many generations of selective breeding, that confer adaptability to local climate conditions and farming systems. For example, high-input wheat production in North-Western Europe is generally based on winter habit varieties that require vernalization, which are mid-late flowering (to escape the risk of late frosts causing sterility), with a semi-dwarf stature (which allows for high grain yields while minimizing the risk of crop losses through lodging). Here, the primary SHW were taller and earlier flowering than ‘Robigus’, with the QTL effects of the alternative SHW alleles also following this trend.

The alternative SHW alleles at *QFt.niab-2D.1*_*wSHWnam*_ and *QFt.niab-2B.1*_*wSHWnam*_ (found across multiple trials) contributed to earlier *Ft*. Flowering time in wheat is a complex trait influenced by interactions between the environment and several genetic pathways, including response to photoperiod and vernalization, as well as additional loci referred to as ‘*earliness *per se’ genes (Cockram et al. 2007). Photoperiod response genes (*Ppd*) play an important role in determining flowering time in wheat (Snape et al. 2001). ‘Robigus’ has wild-type photoperiod sensitive alleles at *Ppd-B1* and *Ppd-D1*, with flowering promoted in long days (Bentley et al. 2013). It is likely that *QFt.niab-2D.1*_*wSHWnam*_ and *QFt.niab-2B.1*_*wSHWnam*_, represent *Ppd-D1* and *Ppd-B1* (respectively), as these genes are located at colinear positions on homoeologous chromosomes 2D and 2B (Law et al. 1978). Furthermore, the peak markers for *QFt.niab-2D.1*_*wSHWnam*_ (14.9 to 33.4 Mb) correspond well with the Chinese Spring reference position for the gene (*TraesCS2D02G079600*, 2D: 34.0 Mb, IWGSC 2018). For the most significant hit for *QFt.niab-2D.1*_*wSHWnam*_, 51 of the primary SHW carried an alternative allele to ‘Robigus’ at the peak marker (AX.94603120), suggesting that alternative haplotypes linked to an earlier flowering response may be common in the *Ae. tauschii* donors. This contrasts with another SHW NAM population in which the *Ppd-D1* haplotype of the recurrent parent (‘Norin 61’) was linked to earlier flowering (Gorafi et al. 2018). Photoperiod-insensitivity may have been incorporated from *T. durum* on the A and/or B genome, although only *Ppd-B1* appears to have been detected in our NAM (as *QFt.niab-2B.1*_*wSHWnam*_). Allelic variation at *Ppd-B1* has been shown to be important in controlling *T. durum* flowering time (Würschum et al. 2019). No QTLs for flowering time were identified consistently in every trial and it is probable that observed variation in *Ft* across trials was caused by different combinations of *T. durum* and *Ae. tauschii* alleles at different genes in the complex flowering time pathway in each nested population.

*QFt.niab-4A.1*_*wSHWnam*_ was found for *Ft* on chromosome 4A across two trials, where peak markers ranged from 681.4 to 735.8 Mb and the alternative SHW alleles gave earlier *Ft*. Genetic control for *Ft* has been found in this region in other wheat multi-founder populations, and work to explore candidate genes is underway [Ian Mackay, personal communication 2022]. The vernalization pathway also plays an important role in controlling *Ft* in wheat, with several major vernalization genes (*Vrn*) characterized (Snape et al. 2001). As the peak markers for *QFt.niab-7D.1*_*wSHWnam*_ ranged from 17.8 to 61.2 Mb on chromosome 7D, it is likely that this is the vernalization gene *Vrn-D3* (*TraesCS7D02G111600*, 7D: 68.4 Mb, IWGSC 2018). Further investigation is needed into the underlying, and potentially, useful novel haplotypes at the QTLs and major gene loci that have been mapped here. Introgression libraries, such as chromosome segment substitution lines (CSSLs; Horsnell et al. 2023), that incorporate specific introgressions from *Ae. tauschii* accessions across the bread wheat genome, could subsequently be used to better understand the function of these potentially novel haplotypes.

### Plant height (Ph)

Dramatic increases in global wheat yields in the latter half of the twentieth century, called the ‘Green Revolution’, were driven in part by changes in plant architecture and physiology. These changes were linked to a set of reduced height (*Rht*) genes. ‘Robigus’ is known to possess the dwarf allele at *Rht-B1* (Gordon et al. 2015). The *Rht-B1* physical position in the reference genome of cv. ‘Chinese Spring’ is located on chromosome 4B at 30.9 Mb (*TraesCS4B02G043100*; IWGSC 2018). A QTL close to this region was found only in the Full21 trial, with a peak marker located at 25.8 Mb (*QPh.niab-4B.1*_*wSHWnam*_, Supplementary Table S5). The dwarf phenotype in *T. durum* is also typically controlled by *Rht-B1* (Subira et al. 2016) and it is likely that a high proportion of tetraploid donors would have also carried the dwarfing allele at this locus; only four nested populations were clearly segregating for the QTL. Due to the back-cross structure of the population, ~ 75% of each genotype’s genome would originate from ‘Robigus’. Therefore, if there are rare alleles in only a small number of primary SHW, statistical power will be low to map these QTLs. *QPh.niab-4B.1*_*wSHWnam*_ was found in the Full21 trial, where most genotypes were screened and statistical power to find rarer QTLs would have been higher than the Core Nurseries. The QTL *QPh.niab-5A.2*_*wSHWnam*_ was significant in the Core18 and the Full21 trial, but just below the significance threshold in the Core17 and Core19 trials. Furthermore, *QPh.niab-5A.1*_*wSHWnam*_ was mapped 60.9 Mb away from *QPh.niab-5A.2*_*wSHWnam*_ in the Core20 trial and may have been the same QTL. There is a known dwarfing gene on chromosome 5A called *Rht12*, although it has been mapped to the distal end of the long arm (Sun et al. 2019). Other studies have identified QTLs for *Ph* on 5A in wheat (Griffiths et al. 2012; Yu et al. 2020) and further work is needed to establish the candidate gene for *QPh.niab-5A.2*_*wSHWnam*_.

The *Ph* QTL *QPh.niab-6A.1*_*wSHWnam*_ was notable for its high significance. There are several known *Rht* genes located on chromosome 6A (McIntosh et al. 2017, 2013), including *Rht-24* which is an important determinant of *Ph* in global winter wheat (Würschum et al. 2017). Tian et al. (2022) used map-based cloning to isolate and identify *Rht-24* as *TraesCS6A02G221900*, located at 413.7 Mb on chromosome 6A in the reference genome (IWGSC 2018). The peak markers and physical position of *QPh.niab-6A.1*_*wSHWnam*_ varied across trials (114.2 to 405.4 Mb on 6A) and the QTL peaks typically extended across large stretches of the pericentromeric region of chromosome 6A. Reduced recombination rates across the physically large pericentromeric region would have decreased mapping resolution. The wide significance peaks of *QPh.niab-6A.1*_*wSHWnam*_ may have also been caused by the effects of multiple loci. *Rht-25* has also been mapped to a region close to the centromere on chromosome 6A (144.0–148.3 Mb; Mo et al. 2018). Furthermore, the Gibberellic Acid-sensitive *Rht* genes *Rht-14*, *Rht-16* and *Rht-18*, that originate from *T. durum* mutants, have been mapped to the short arm of chromosome 6A (Haque et al. 2011). Although when the peak marker of *QPh.niab-6A.1*_*wSHWnam*_ was used as a covariate in an additional scan using the Full21 trial, no other hits were observed on chromosome 6A. *QPh.niab-6D.1*_*wSHWnam*_ was found in three trials and may have been a D genome homoeologue for one of these 6A *Rht* genes. Other QTLs linked to plant height have been identified on 6D in previous studies (Wang et al. 2020), although *Ph* QTLs have been found distributed across every chromosome in wheat (Mao et al. 2010). The peak marker for *QPh.niab-6D.1*_*wSHWnam*_ (AX.94940605) was the only significant marker on 6D and could have been anchored to the wrong chromosome. However, there were no alternative BLAST hits on 6A, suggesting the marker was not linked to *QPh.niab-6A.1*_*wSHWnam*_. Further efforts are needed to explore the haplotype diversity of the 6A centromeric regions in the NIAB_WW_SHW_NAM population to examine the genetic control of *Ph* and narrow down the *QPh.niab-6A.1*_*wSHWnam*_ genetic interval.

### Yellow rust infection (Yr)

Primary SHW are recognized as promising resources for resistance to yellow rust and other pathogens (Li et al. 2018). Yellow rust infection (*Yr*) was scored in three field trials, and in each of these experiments QTL *QYr.niab-4D.1*_*wSHWnam*_ was the most significant hit: 37 primary SHW donors carried the alternative allele to ‘Robigus’ that was linked to improved resistance. The primary SHW donors had a lower mean *Yr* compared to ‘Robigus’ in the Full21 trial (Fig. 3c). The peak marker for *QYr.niab-4D.1*_*wSHWnam*_ (AX.94546744, located at 1.4 Mb on chromosome 4D) was identical across trials. *Yr28* is a previously described *Ae. tauschii* resistance gene on the short arm of chromosome 4D (Singh et al. 2000). Additionally, a gene originating from *Ae. tauschii* and designated as *YrAS2388* has been mapped to the same chromosome arm (Huang et al. 2011). *Yr28* and *YrAS2388* are now considered to be the same gene (Liu et al. 2013). Athiyannan et al. (2022) showed that *YrAS2388* and another source of resistance found in an *Ae. tauschii* accession from Turkmenistan (*YrAet672*) were haplotype variants of *Yr28*. These haplotype variants encode nucleotide-binding leucine-rich repeat proteins typical of plant disease resistance genes (Athiyannan et al. 2022; Zhang et al. 2019). The susceptible allele of *YrAS2388* in the reference genome of cv. ‘Chinese Spring’ is located in the region of 4D:1,821,950-1825589 bp (IWGSC 2018; Zhang et al. 2019). The peak hit for our yellow rust resistance QTL *QYr.niab-4D.1*_*wSHWnam*_ was ~ 400 kb away from this location, indicating that *Yr28* is a very strong candidate for *QYr.niab-4D.1*_*wSHWnam*_. There is limited evidence to suggest that *Yr28* has been used as a source of resistance in modern wheat (Athiyannan et al. 2022; Zhang et al. 2019). Here, we propose that *Yr28* can be effectively deployed in a UK winter wheat background (‘Robigus’) to confer resistance against current yellow rust pathotypes under field conditions in a maritime, temperate climate. In nested populations where *Yr28* was segregating, *Yr* resistance was actively selected for by breeders. Furthermore, *Yr28* is reported to perform better at warmer field temperatures (Singh et al. 2000), and so could be a useful source of resistance for future climate scenarios.

### Breeder selection

The primary aim of SHW creation was to capture novel D sub-genome diversity, explaining the disparity between the number of unique *Ae. tauschii* (47) compared to *T. durum* (15) accessions captured. The majority of SHW used in developing the NIAB_WW_SHW_NAM used ‘Hoh-501’ as the tetraploid because it gave a high success rate during resynthesis. However, there was indication that breeders selected toward other *T. durum* backgrounds in the Core Nurseries. For example, ‘Hoh-501’ was a component of a portion of SHW-derivatives in every Core Nursery, but ‘UKR-OD 1530.94’, which was a component of some SHW-derivatives grown in just the Core20 and Core21 trials, had a much higher selection proportion by the breeders. With higher representation of L2 *Ae. tauschii* donors in the SHW, it was unsurprising that L2 derived genotypes had a higher proportion of selection by breeders compared to L1. Furthermore, in the Core19 and Core20 trials only one nested population with L1 parentage was present in the trial, which could have biased the selection. A selection experiment with more evenly distributed *Ae. tauschii* and *T. durum* backgrounds across trials would be needed before drawing statistically-backed conclusions on breeder selection patterns in the SHW-derived genotypes.

SHW-derivatives are known to be a valuable resource for breeding toward improved grain yield (Jafarzadeh et al. 2016), which, when combined with their rich allelic diversity, makes them appealing candidates for improving the genetic gain of breeding programs. In addition to their use in genetic mapping, the Core Nursery field experiments were intended to provide wheat breeders access to a genetically diverse pre-breeding resource in an adapted European winter wheat background. Breeders selected a high proportion—between 21 and 58%—of the genotypes in each Core Nursery for further assessment. Once genotypes enter commercial breeding programs, their impact can be difficult to track. However, NIAB SHW-derived material continues to be evaluated in commercial breeding nurseries for a range of novel and standard traits: the breeding company DSV have recently developed a variety based on crosses featuring NIAB SHW genetics (Matt Kerton, DSV, personal communication). The breeders selected for *Yr* resistance in the Core Nurseries with reduced fungicide application (Core17 and Core18). Furthermore, in nested populations that were segregating for *QYr.niab-4D.1* across the Core Nurseries, there was a difference between the frequency of the SHW *QYr.niab-4D.1* allele linked to improved resistance in genotypes selected by at least one breeder compared to those not selected; 33% of selected genotypes (202/606) carried the resistance allele, compared to 21% (208/980) in the unselected genotypes. These observations highlight that the resistance provided by *Yr28* was favored by breeders and further work is needed to establish if this source of resistance is effective in other genetic wheat backgrounds.

## Concluding remarks

SHW-derived wheat pre-breeding genotypes offer the potential to characterize and introduce novel diversity into wheat research and breeding. The NIAB_WW_SHW_NAM population is a large resource that captures structured diversity from a collection of SHW lines developed using a diverse range of *T. durum* and *Ae. tauschii* donors. The population is a valuable mapping resource for the detection of potentially useful genetic regions, such as *QYr.niab-4D.1*_*wSHWnam*_ which is a candidate for *Yr28*. This resource has been generated in an adapted UK winter wheat background, making it a useful pre-breeding resource—as evidenced in practice by uptake of promising genotypes by commercial breeders. Therefore, it is likely the NIAB_WW_SHW_NAM population complements and adds to the available resources for increasing genetic diversity in European wheat.

### Supplementary Information

Below is the link to the electronic supplementary material.

Supplementary file1 (PDF 12409 kb)

Supplementary file2 (XLSX 119 kb)
