# Supplementary material for: A new winter wheat genetic resource harbors untapped diversity from synthetic hexaploid wheat
Source: Theor Appl Genet. 2024 Mar 7;137(3):73. doi: 10.1007/s00122-024-04577-1 (PMC10920491; doi:10.1007/s00122-024-04577-1)
Supplement: Supplementary file 1 — Supplementary file1 (PDF 12409 kb) [file 122_2024_4577_MOESM1_ESM.pdf]

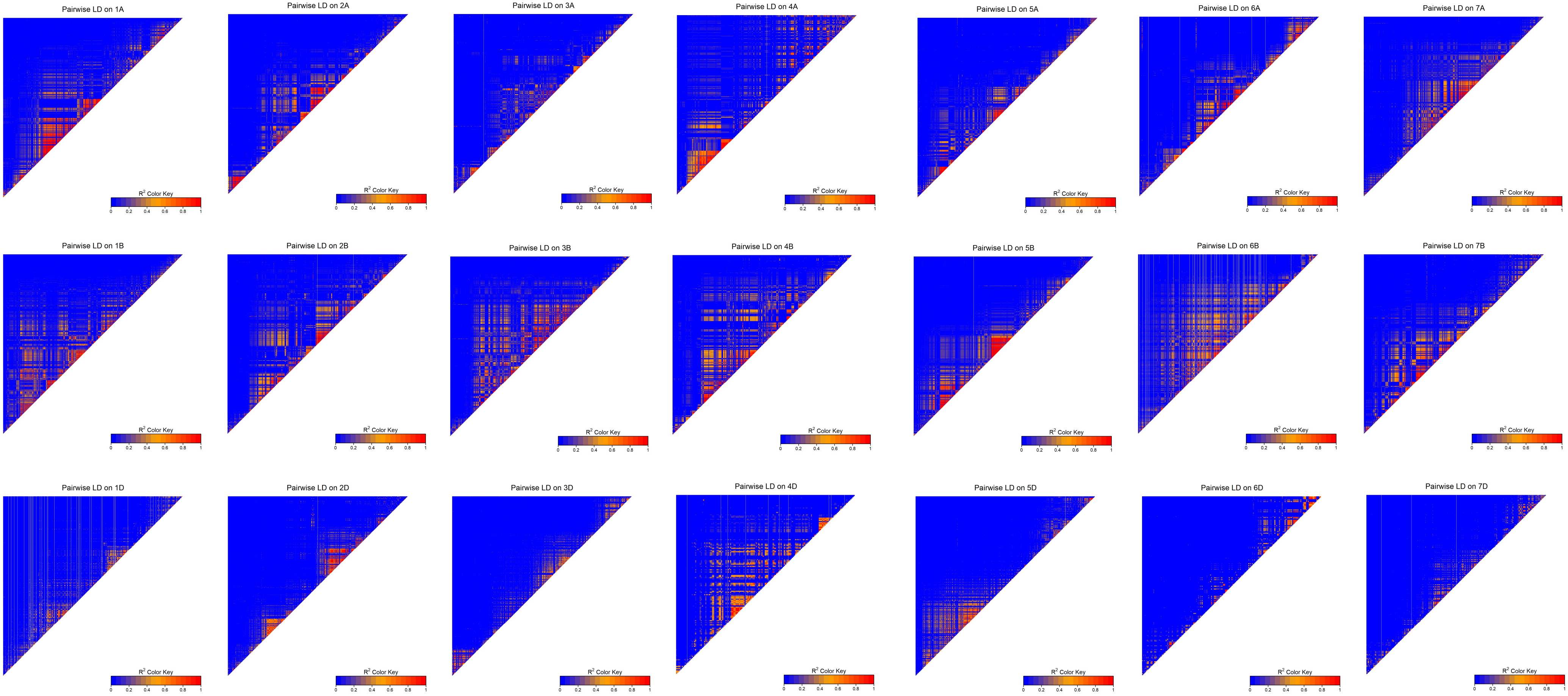

Supplementary Figure S1 – Linkage disequilibrium (LD) between SNPs on each chromosome using 11,074 anchored genetic markers. LD was measured using pairwise correlation between markers on each chromosome (R^2). The LD plots were formed using the R package LDheatmap (Shin et al. 2006).

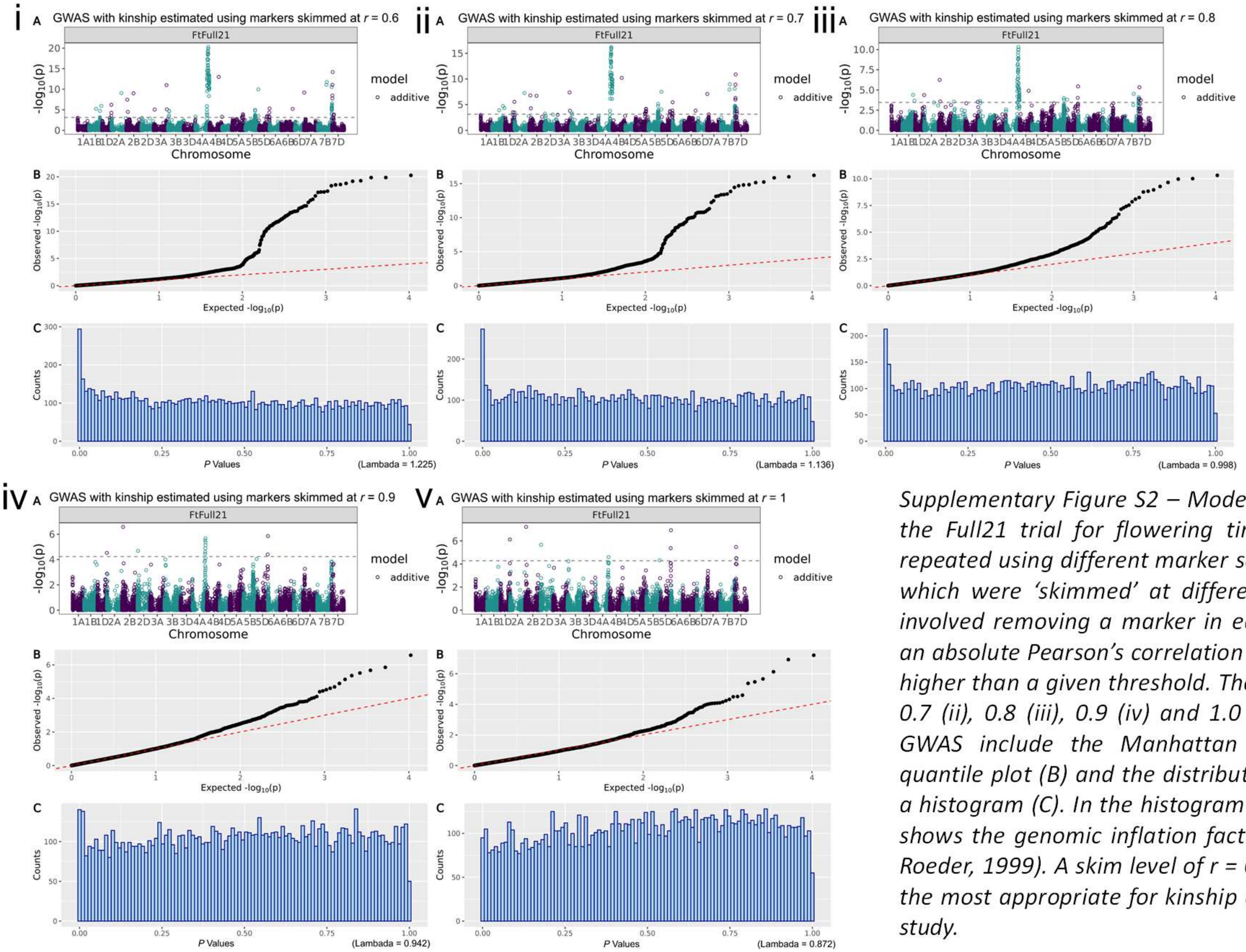

Supplementary Figure S2 – Model testing using data from the Full21 trial for flowering time (Ft). The GWAS was repeated using different marker sets for kinship estimation which were 'skimmed' at different thresholds. Skimming involved removing a marker in each pair of markers with an absolute Pearson's correlation coefficient (r) equal to or higher than a given threshold. The thresholds were: 0.6 (i), 0.7 (ii), 0.8 (iii), 0.9 (iv) and 1.0 (v). The results for each GWAS include the Manhattan plot (A), the quantile-quantile plot (B) and the distribution of P values shown as a histogram (C). In the histogram plots (C), the x axis label shows the genomic inflation factor (Lambada; Devlin and Roeder, 1999). A skim level of r = 0.8 was selected as being the most appropriate for kinship estimation in the present study.

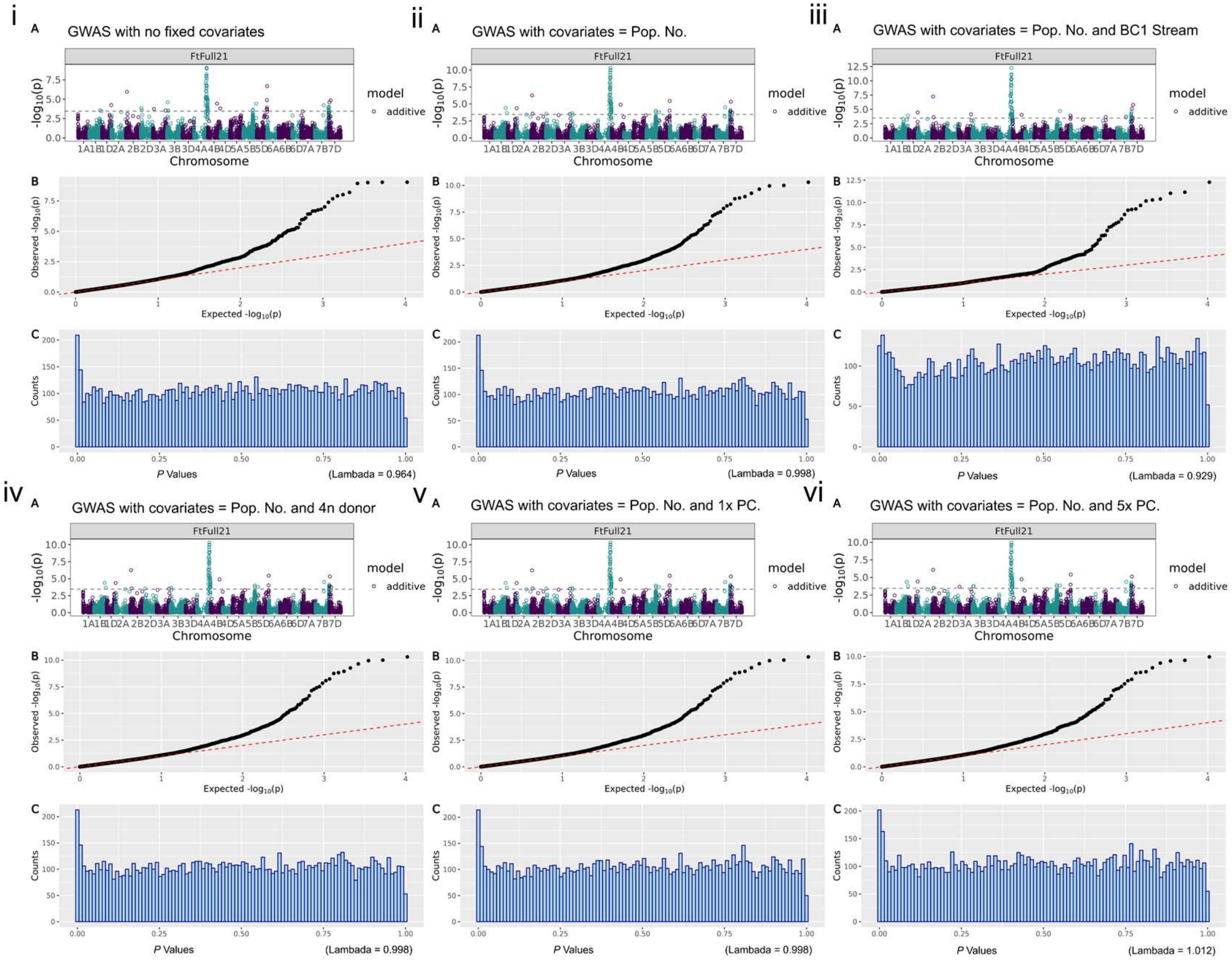

Supplementary Figure S3 – Model testing using data from the Full21 trial for flowering time (Ft). The GWAS was repeated using different fixed covariates, including: no fixed covariates (i), nested-population number (ii), nested-population number and BC1 stream number (iii), nested-population number and tetraploid donor (4n) parentage (iv), nested-population number and a single principal component explaining population structure of the genotypes (v) and nested-population number and 5 principal components (vi). For each GWAS a kinship correction was also included, with markers 'skimmed' with r = 0.8. The results for each GWAS include the Manhattan plot (A), the quantile-quantile plot (B) and the distribution of P values shown as a histogram (C). In the histogram plots (C), the x axis label shows the genomic inflation factor (Lambada; Devlin and Roeder, 1999). The GWAS with the covariate structure of just nested-population number (in addition to the kinship correction) was selected as being the most appropriate for genetic mapping in the present study.

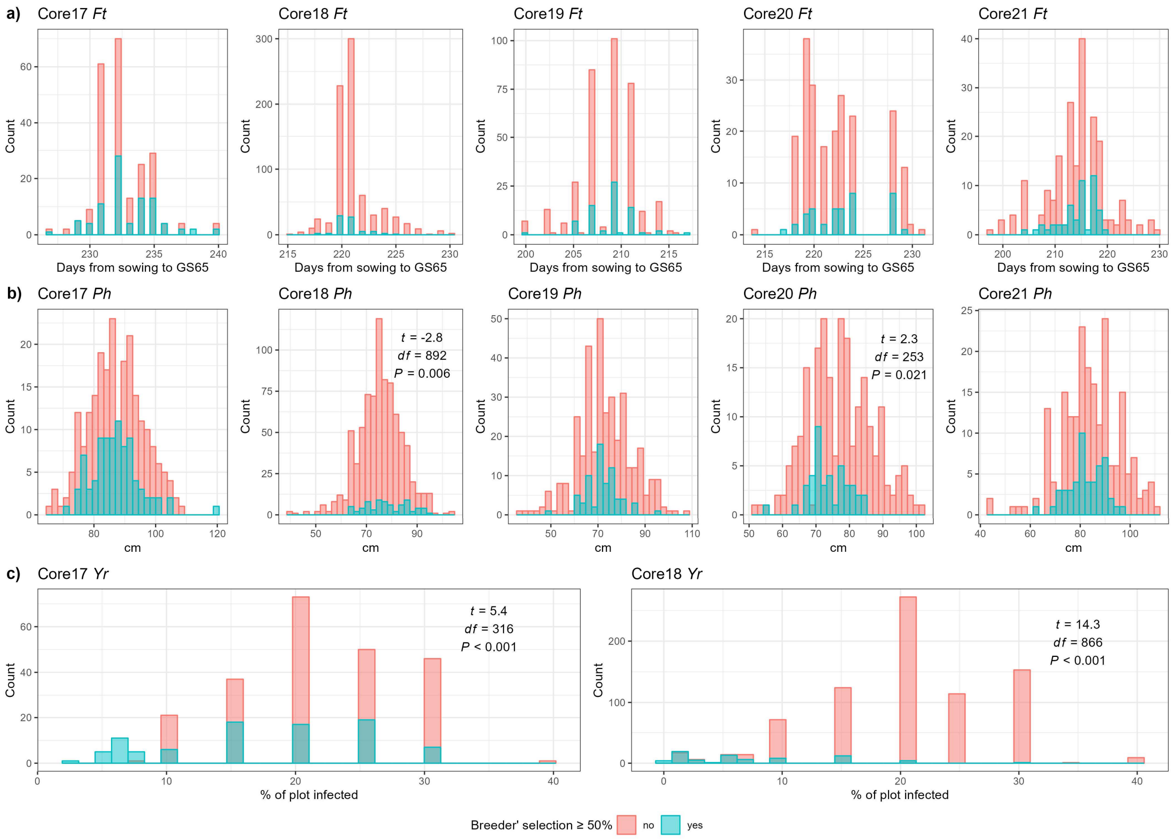

Supplementary Figure S4 – Trends of breeders' selection based on three traits across the Core Nurseries: a) flowering time (Ft), b) plant height (Ph) and c) yellow rust infection (Yr). Genotypes were treated as selected if ≥ 50% of breeders selected the genotype. Where there were significant differences between means of selected versus non-selected genotypes (P < 0.05), t-test results are overlaid on each plot.

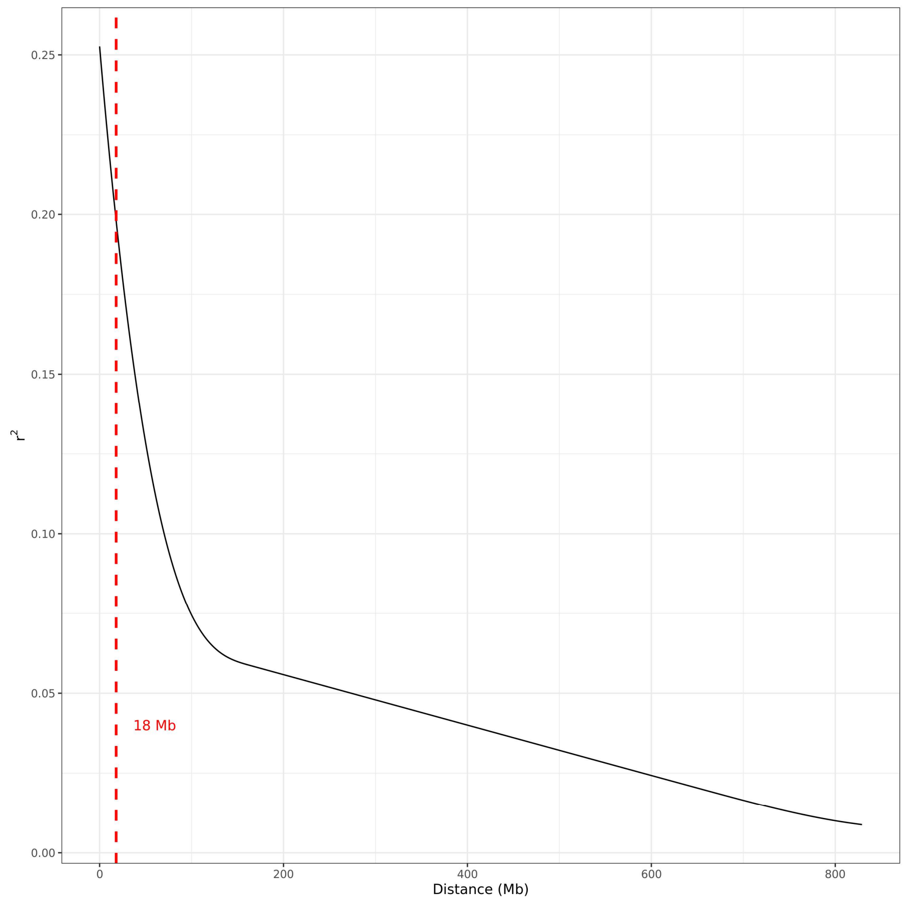

Supplementary Figure S5 – Pairwise correlations (R^2) between markers plotted against the physical distance between those same markers (Mb). Marker data were from the Full21 trial. The plot was formed using the 'LD.plot' function of the R package GWASpoly (Rosyara et al. 2016). This plot was used to determine the window of LD decay at a critical value (R^2 = 0.2) was 18 Mb.

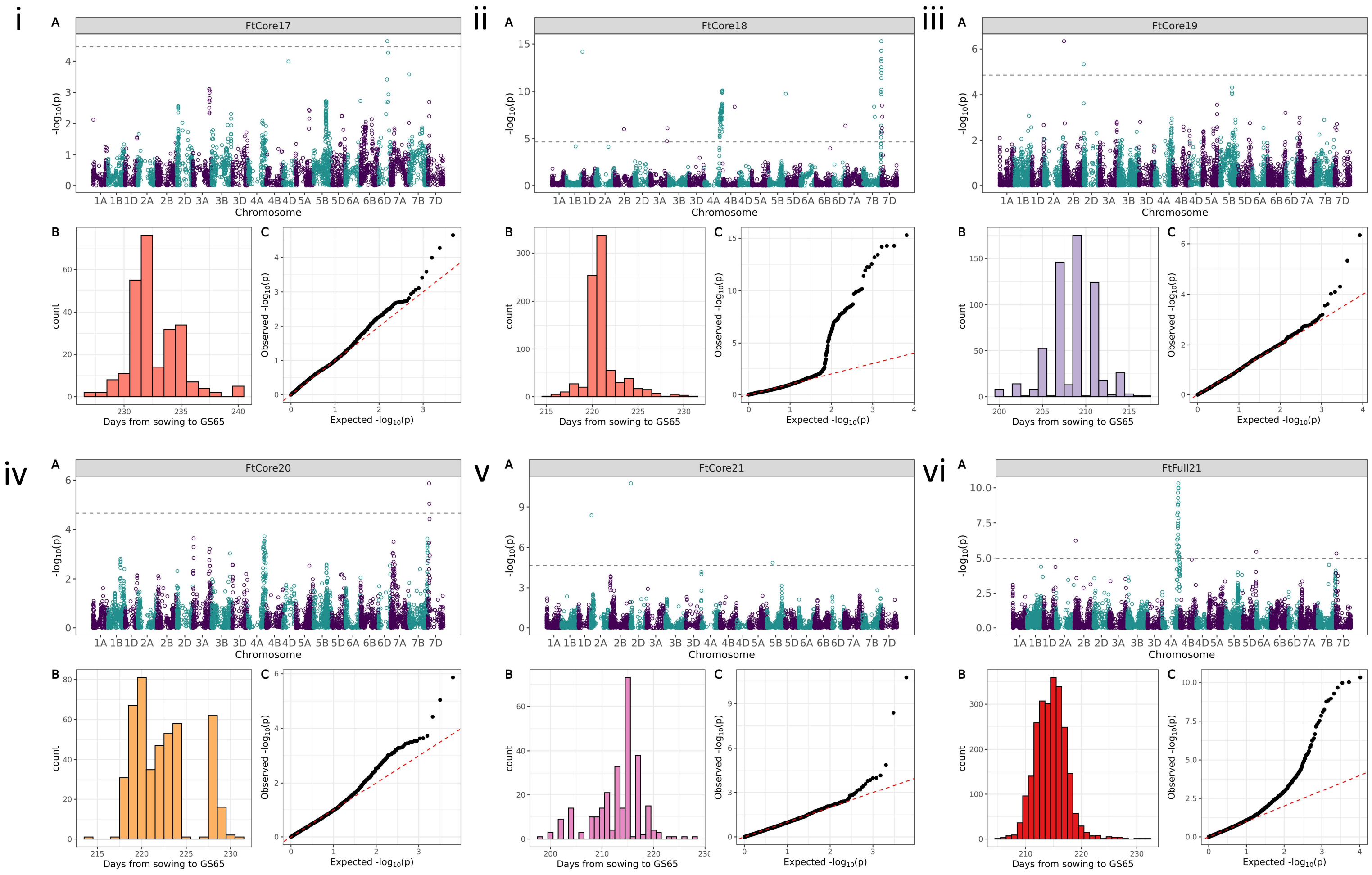

Supplementary Figure S6 – Flowering time (Ft) QTL scan results for each trial: Core17 (i), Core18 (ii), Core19 (iii), Core20 (iv), Core21 (v) and Full21 (vi). Each plot shows a Manhattan plot (A), histogram of the trait distribution for the mapping individuals (B) and a quantile-quantile plot (C).

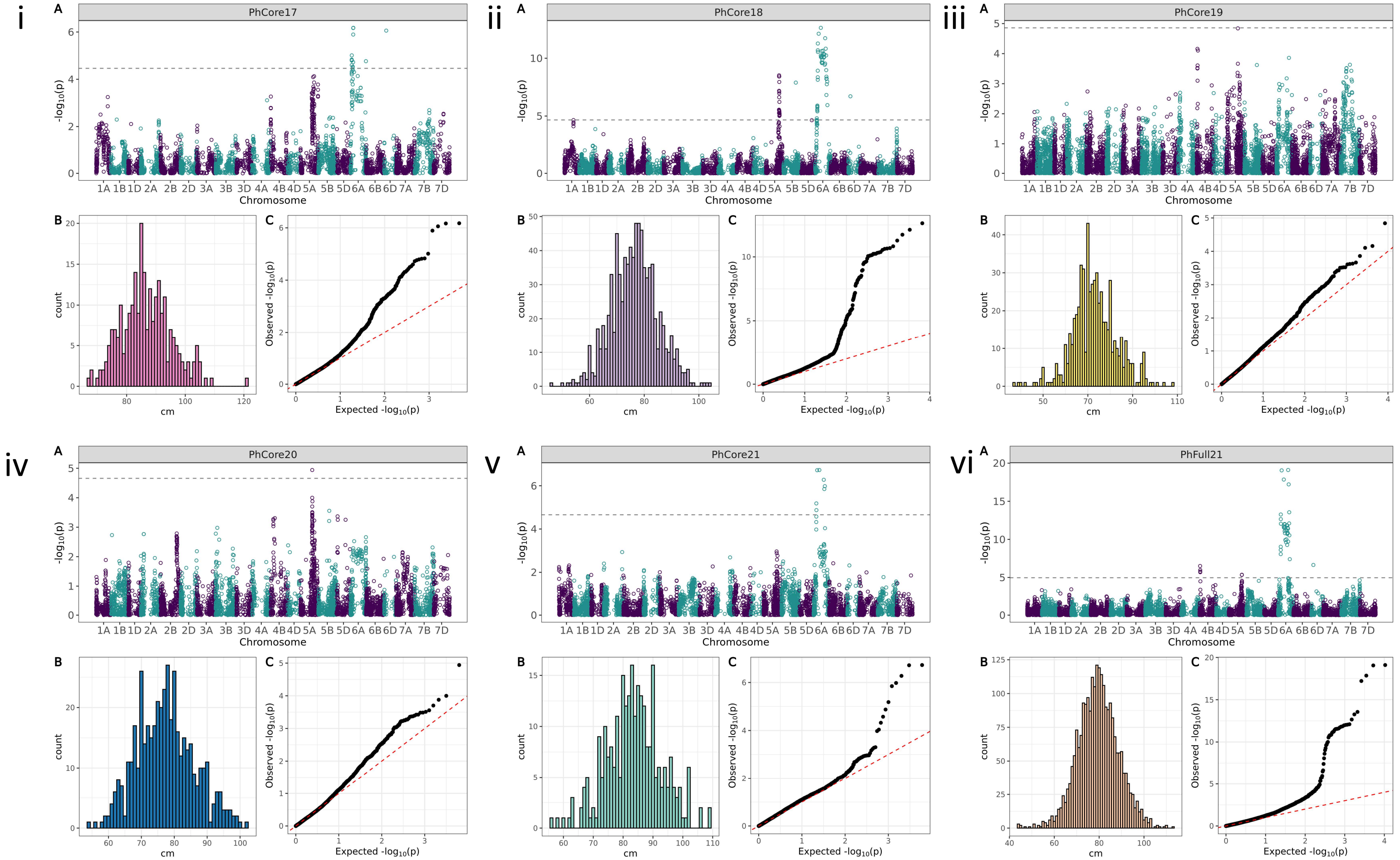

Supplementary Figure S7 – Plant height (Ph) QTL scan results for each trial: Core17 (i), Core18 (ii), Core19 (iii), Core20 (iv), Core21 (v) and Full21 (vi). Each plot shows a Manhattan plot (A), histogram of the trait distribution for the mapping individuals (B) and a quantile-quantile plot (C).

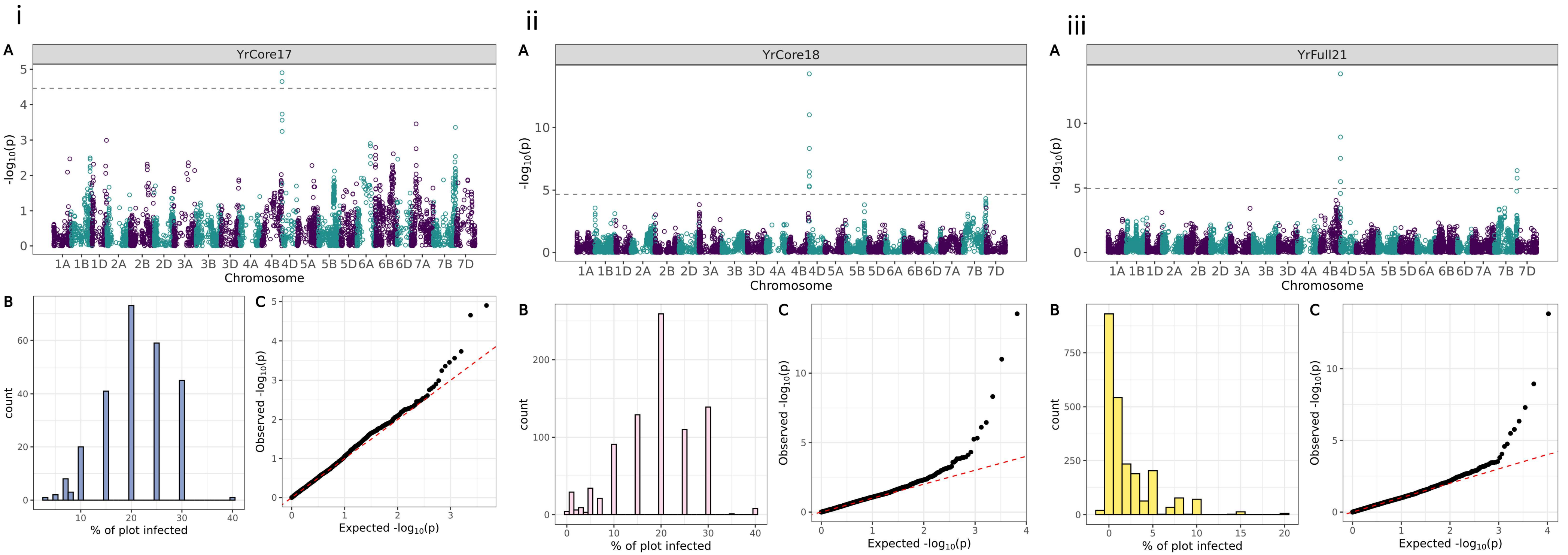

Supplementary Figure S8 – Yellow rust infection (Yr) QTL scan results for the trials: Core17 (i), Core18 (ii) and Full21 (iii). Each plot shows a Manhattan plot (A), histogram of the trait distribution for the mapping individuals (B) and a quantile-quantile plot (C).

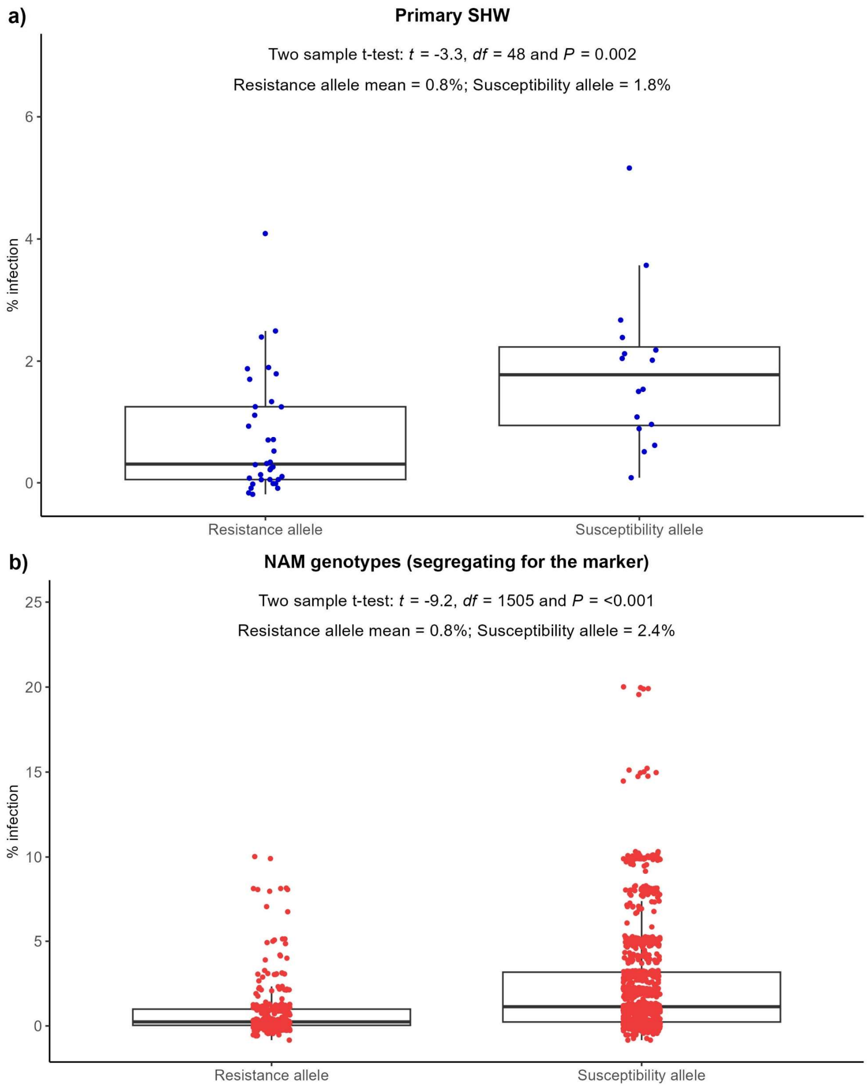

Supplementary Figure S9 – Yellow rust infection (Yr) of genotypes that differ in alleles for the peak SNP (AX.94546744) of QYr.niab-4D.1, which was found in all trials where Yr was assessed (Core17, Core18 and Full21). The 'resistance allele' represents the homozygous alternative SHW genotype which was linked to improved Yr resistance, compared to the homozygous 'Robigus' genotype that showed Yr susceptibility ('susceptibility allele'). Data are from the Full21 trial for: a) 50 of the primary Synthetic Hexaploid Wheat (SHW) parents and b) 1507 genotypes from 32 nested populations clearly segregating for the marker. Two-sample t-test results and sample means are shown for each plot.

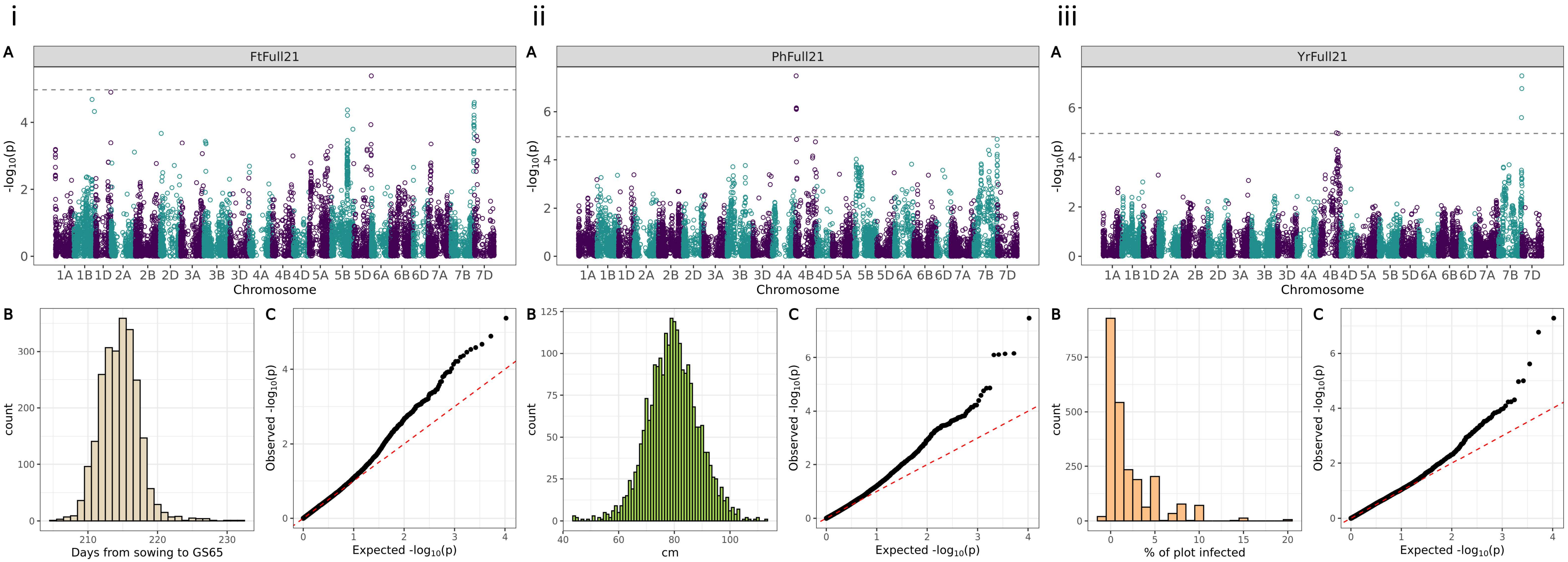

Supplementary Figure S10 – QTL scan results for each trait in the Full21 trial, with QTL found in both the Full21 trial and at least one other trial used as covariates. For flowering time (Ft, i) the QTLs used as covariates were: QFt.niab-2B.1, QFt.niab-4A.1 and QFt.niab-7D.1. For plant height (Ph, ii) the QTLs used as covariates were: QPh.niab-5A.2, QPh.niab-6A.1 and QPh.niab-6D.1. For yellow rust infection (Yr, iii), QYr.niab-4D.1 was used as the only QTL covariate. For the covariate scans, the same model parameters were used as the initial QTL mapping.
